# Poly(C)-binding Protein 2 Regulates the p53 Expression via Interactions with the 5′-Terminal Region of p53 mRNA

**DOI:** 10.3390/ijms222413306

**Published:** 2021-12-10

**Authors:** Damian M. Janecki, Agata Swiatkowska, Joanna Szpotkowska, Anna Urbanowicz, Martyna Kabacińska, Kamil Szpotkowski, Jerzy Ciesiołka

**Affiliations:** Institute of Bioorganic Chemistry, Polish Academy of Sciences, 61-704 Poznan, Poland; djanecki@ibch.poznan.pl (D.M.J.); jsroka@ibch.poznan.pl (J.S.); aniau@ibch.poznan.pl (A.U.); mkabacinska@ibch.poznan.pl (M.K.); kamilsz@ibch.poznan.pl (K.S.)

**Keywords:** p53 protein, poly(C)-binding proteins, p53 mRNA, translation

## Abstract

The p53 protein is one of the major transcriptional factors which guards cell homeostasis. Here, we showed that poly(C)-binding protein 2 (PCBP2) can bind directly to the 5′ terminus of p53 mRNA by means of electrophoretic mobility shift assay. Binding sites of PCBP2 within this region of p53 mRNA were mapped using Pb2+-induced cleavage and SAXS methods. Strikingly, the downregulation of PCBP2 in HCT116 cells resulted in a lower level of p53 protein under normal and stress conditions. Quantitative analysis of p53 mRNA in PCBP2-downregulated cells revealed a lower level of p53 mRNA under normal conditions suggesting the involvement of PCBP2 in p53 mRNA stabilisation. However, no significant change in p53 mRNA level was observed upon PCBP2 depletion under genotoxic stress. Moreover, a higher level of p53 protein in the presence of rapamycin or doxorubicin and the combination of both antibiotics was noticed in PCBP2-overexpressed cells compared to control cells. These observations indicate the potential involvement of PCBP2 in cap-independent translation of p53 mRNA especially occurring under stress conditions. It has been postulated that the PCBP2 protein is engaged in the enhancement of p53 mRNA stability, probably via interacting with its 3′ end. Our data show that under stress conditions PCBP2 also modulates p53 translation through binding to the 5′ terminus of p53 mRNA. Thus PCBP2 emerges as a double-function factor in the p53 expression.

## 1. Introduction

The p53 protein is one of the major transcription factors responsible for cell homeostasis and cell stress-response [1,2]. It has been well documented that most mutations in the *TP53* gene result in cancer development [3]. Recent research focuses on how the expression of p53 protein is modulated and connected to other processes in the cell [4]. It emerges that regulation of the p53 expression is based on an intricate network of direct and indirect interactions of many protein and nucleic acid factors with the *TP53* gene, p53 protein and p53 mRNA. One of such regulatory mechanisms depends on the interactions of selected proteins with p53 mRNA, particularly with its 5′ and 3′ ends [5,6]. For example, the 3′-terminal region of p53 mRNA is recognised by protein human antigen R (HuR) which stabilises p53 mRNA and enhances p53 synthesis under UV treatment [5,7]. Cytoplasmic polyadenylation element-binding protein 1 (CPEB1) seems to stimulate p53 translation since its knockdown causes a decrease in p53 amount [8]. On the other hand, the 5′-terminal region of p53 mRNA has also been shown to bind to several proteins, such as polypyrimidine tract-binding protein (PTB), SFPQ protein (also known as PSF), death-associated protein 5 (DAP5), nucleolin and ribosomal protein RLP26 [5,9]. Thus, interactions of the 5′-terminal region of p53 mRNA with protein factors seem to be crucial for the regulation of p53 expression. 

Recently, we have applied RNA-centric chromatography combined with mass spectrometry analysis to identify new proteins which can modulate the p53 expression via interacting with the 5′-terminal region of p53 mRNA [6]. We have demonstrated that one of the proteins involved in the regulation of p53 transcription and potentially translation is the heterogeneous nuclear ribonucleoprotein K (hnRNP K) which belongs to the family of poly(C)-binding proteins (PCBP) [6]. It has previously been proposed that p53 stimulates hnRNP K at the transcriptional level [10] and both proteins act together to activate p53 downstream genes upon stress conditions [11]. Our research showed that hnRNP K and p53 do not only work together to activate the p53 downstream genes but they also influence each other at the transcriptional level. Additionally, hnRNP K is likely to stimulate p53 synthesis via interaction with the 5′ terminus of p53 mRNA under specific conditions [6]. 

The poly(C)-binding proteins family includes two subsets of proteins present in mammalian cells: hnRNP K and PCBP (1–4). PCBPs are abundantly expressed in mammalian cells and are estimated to constitute approximately 0.5% of total proteins [12]. These proteins share a common feature, namely the presence of three hnRNP K homology domains (KH 1–3) composed of approximately 70 amino acids each [13]. The KH1 and KH3 domains—but not the KH2 domain—have been shown to bind RNA [14,15]. Moreover, the three KH domains together bind RNA synergistically whereas, for comparison, a single KH domain binds RNA weakly [15,16].

Here, we focused on PCBP2, identified in our earlier studies [6] as being able to bind to the 5′-terminal region of p53 mRNA. It has earlier been proposed that PCBP2 together with PCBP1 is involved in the enhancement of mRNA stability via interaction with C-rich elements within mRNAs [17]. PCBP2 and DHX30 have been identified as interactors with CG-rich motifs present in 3′ UTRs of mRNAs that are involved in a translational control mechanism [18,19]. It has also been shown that PCBP2 enhances the translation of poliovirus RNA by binding to its 5′ noncoding region [20,21,22]. Moreover, recent research has demonstrated the PCBP2’s engagement in the regulation of p73 expression and antioxidant response, neural apoptosis and also in the migration and invasion of glioma cells [23,24,25]. 

In the present work, we showed the direct interactions of PCBP2 with the 5′-terminal region of p53 mRNA and structural variants of this region, applying the EMSA method. Subsequently, we mapped the PCBP2 binding sites using Pb2+-induced cleavage and small-angle X-ray scattering (SAXS) methods. These approaches enabled us to visualise the interaction sites on the secondary and 3D structures of RNA, respectively. To find out the potential role of PCBP2 in the regulation of p53 expression we analysed the impact of the PCBP2 depletion or overexpression on the p53 protein and mRNA levels under normal and stress conditions in the HCT116 cell line. Finally, we treated PCBP2-overexpressed cells with rapamycin and a mix of rapamycin and doxorubicin to gain more details about the PCBP2 regulatory function for p53 translation. 

## 2. Results and Discussion

### 2.1. PCBP2 Binds Directly to the 5′-Terminal Region of p53 mRNA In Vitro

**Figure 1:**
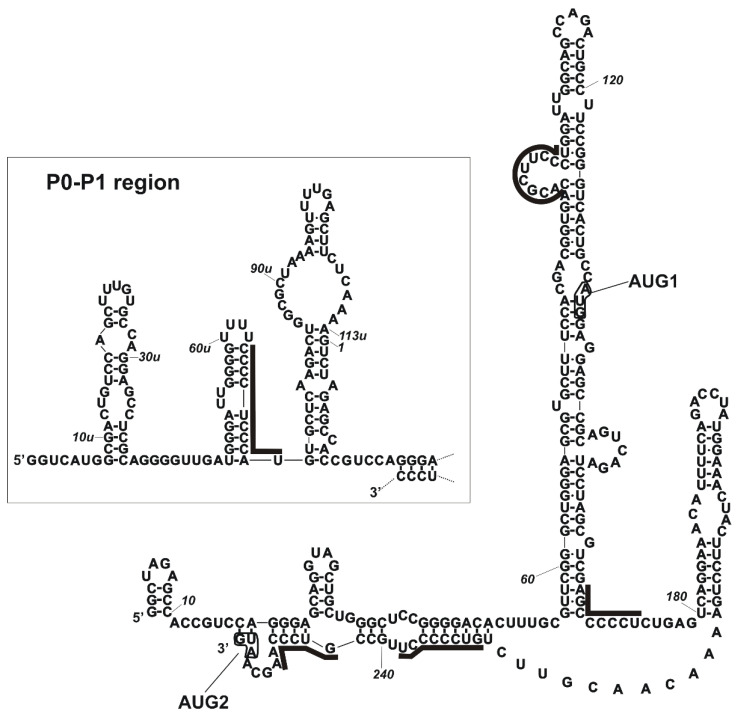
The predicted binding sites for PCBP2 are displayed in the secondary structure model of the 5′-terminal region of p53 mRNA. Binding sites were defined based on the ATtRACT database and literature information [9,26]. Binding sites for PCBP2 are marked by continuous lines (GCCCC, UCCCC, UUCCC, CCCCC, AGCCC, GUCCC, CCCCU, CCCUCCC, CCUCCCA); The mRNA region which spans the transcription initiation sites P0 and P1 are shown in the inset.

Our latest study has shown that hnRNP K protein can bind directly to the 5′-terminal region of p53 mRNA and it influences the p53 expression particularly under stress conditions [6]. As hnRNP K belongs to the family of poly(C)-binding proteins (PCBPs), we wondered whether others protein members of that family could affect the p53 expression profile, particularly via interaction with the 5′ terminus of p53 mRNA. Importantly, our data obtained from RNA-centric affinity chromatography combined with a mass spectrometry analysis revealed that PCBPs were potential candidates interacting with the 5′-terminal region of p53 mRNA [6]. We identified PCBP1, PCBP2, PCBP3 in all applied cell lines (MCF-7, HepG2 and HT-29) whereas PCBP4 was only found in HT-29 cells [6]. Since PCBP2 was among the top 30 proteins with the highest MS score and it has been shown to bind to the 5′ terminus of viral and cellular mRNAs we decided to focus on this protein in our further analysis. PCBP2 was characterised by a similar number of sequence matches and high sequence coverage when using both variants of the 5′ terminus of p53 mRNA, P0-Δ40p53 and P1-Δ40p53 under all tested conditions (Table 1). We did not observe any significant differences in peptide numbers when comparing the no-stress conditions and the stress conditions generated by doxorubicin [6]. The analysis of the potential PCBP2 binding sites within P0-Δ40p53 and P1-Δ40p53 RNAs showed at least a few poly(C) tracts that might be recognised by this protein (Figure 1 and [6,9]). 

Earlier studies have shown that the binding of PCBPs to RNA relies on the presence of short tracts of cytosine stretches (reviewed in [13]). Later, in vitro SELEX experiments have revealed that high-affinity binding of hnRNP K requires a single stretch of Cs while the PCBP2 binding site encompasses three such short stretches of 3–5 nucleotides in length. The stretches were flanked by A- and U-rich segments with a strong bias against G residues. The spacing between C stretches was variable but tended to be short [26]. Recently, the binding specificities of a different set of RNA-binding proteins (RBPs) have been analysed by a high-throughput assay [27]. RBPs bind a small subset of the sequence space. In particular, the top motif identified for hnRNP K consists of three Cs flanked by G and A at the 5′ and 3′ sides. Three-Cs top motifs also prevail for PCBP1 and PCBP2, with much less specified flanking residues. Proteins with KH domains tend to prefer large hairpin loops and have a preference for specific flanking bases and bipartite motifs, suggesting that recognition of longer stretches of RNA by multiple KH domains may be common [27]. 

The contributions of separate KH domains of PCBP1 to binding of C-rich oligonucleotides have also been analysed showing that KH1 makes the most stable interactions with both RNA and DNA, KH3 binds with intermediate affinity and KH2 interacts only with DNA [15]. The crystal structure of KH1 bound to a 5′-CCCTCCCT-3′ DNA oligomer shows a 2:1 complex stoichiometry with two protein molecules bound to immediately adjacent oligonucleotide target sites. Additional experiments have revealed that C residues are preferred at all four positions in the oligonucleotide binding cleft and that a C-tetrad binds to KH1 with 10 times higher affinity than a C-triplet (Kds of 33 μM and 3.5 μM were determined for 10-nucleotide-long oligomers with a single binding site) [15].

To demonstrate that PCBP2 can directly interact with the 5′ terminus of p53 mRNA, the electrophoretic mobility shift assay (EMSA) was performed for two length variants of the 5′ terminus of p53 mRNA, P1-Δ40p53 and P0-Δ40p53 RNAs. These variants are a consequence of the existing P1 and P0 transcription promoters of the *TP53* gene. It has been observed that p53 transcripts start from P1 promoter mostly in carcinoma cells while P0-initiated transcripts prevail in healthy cells [28]. Recently, we have shown that the 5’-terminal regions of p53 mRNA variants influence the p53 expression pattern at the translational level [29,30]. Importantly, the 5’-terminal region of the p53 transcript is also a docking platform for protein factors that can adjust p53 expression to changes in the cell environment. However, only a few proteins interacting with this region of p53 mRNA have been found so far to act as p53 regulators at the translational level [5,31].

**Figure 2:**
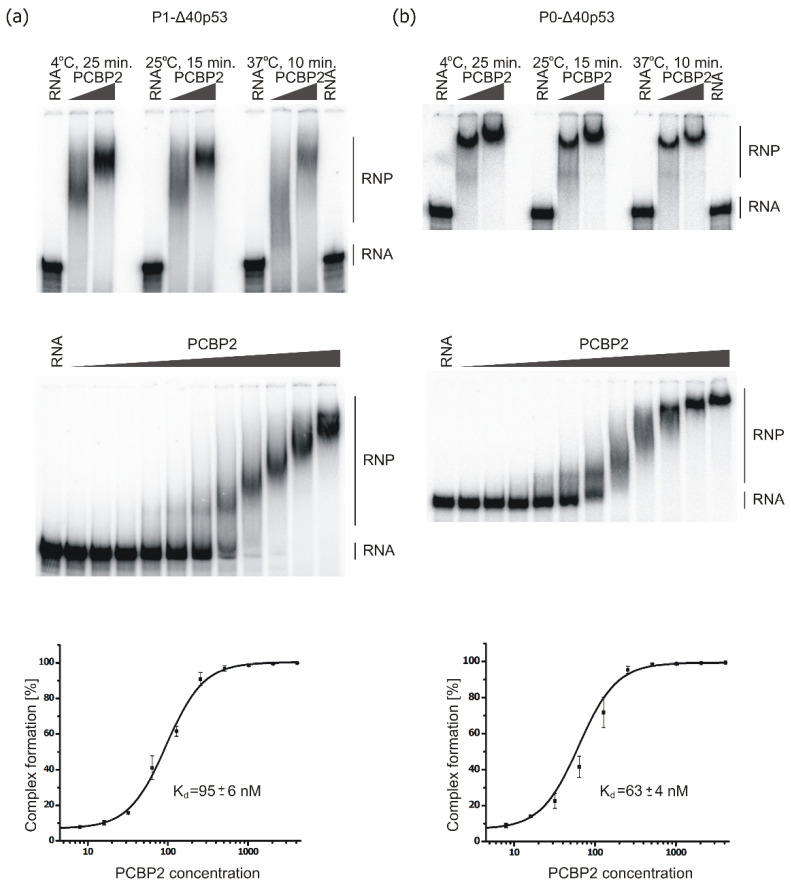
PCBP2 interacts directly with P1-Δ40p53 RNA (**a**) and P0-Δ40p53 RNA (**b**) in in vitro conditions. The EMSA assays were performed by incubation of [32P]-labelled RNAs with two final concentrations of purified PCBP2: 1000 nM and 2500 nM under three different conditions specified in the figure (top panel) and then with increased concentrations of PCBP2 3.9, 7.8, 15.6, 31.3, 62.5, 125, 250, 500, 1000, 2000 and 4000 nM at 4 °C for 25 min (middle panel). The samples were separated on native 4% polyacrylamide gels. The first lane in each EMSA panel represents the RNA oligomer incubated without the protein. Unbound RNAs and RNP complexes are indicated. Graphs representing dissociation constant (Kd) curves of PCBP2 binding to P1Δ40p53 and P0Δ40p53 RNAs, respectively, measured from three independent experiments are also shown (bottom panel).

The EMSA experiments were performed using PCBP2 which was overexpressed in *E. coli* as a fusion protein and then, the protein was purified using nickel affinity chromatography (Figure S1). Afterwards, radiolabelled RNAs were incubated with the purified PCBP2 under three different conditions to confirm binding of PCBP2 to the 5′-terminal region of p53 mRNA (Figure 2a,b, upper panels). In all tested conditions, we observed shifted bands which indicate the formation of RNA-protein complexes. The shifted bands were smeared, suggesting that more than one protein molecule may bind to the RNA. Similar effects were observed for complexes of hnRNP K with P1-Δ40p53 and P0-Δ40p53 RNAs [6]. Next, we performed EMSA at increasing concentrations of PCBP2 to calculate the dissociation constant values, Kds (Figure 2a,b, middle panels). Assuming a sigmoidal fit model, for both complexes PCBP2—P1-Δ40p53 RNA and PCBP2—P0-Δ40p53 RNA, the Kds were 95 ± 6 nM and 63 ± 4 nM, respectively (Figure 2a,b, lower panels). 

It has been reported that the concentration of PCBP2 in cells is approximately 100 nM [32]. For the native *α*-globin 3′UTR with unfractionated cell extract, the apparent Kd as low as 0.5 nM has been determined [33]. However, the recombinant PCBP2 variant binds to the 3′UTR with Kd of 20 nM and in vitro selected RNAs with 10–20-fold higher binding affinity [26]. For the complex of PCBP2 with c-myc IRES RNA, Kd of 160 nM has been estimated [34]. Since PCBP proteins have three KH motifs, their individual contribution to RNA binding is of particular interest. A respective analysis has been performed for separate domains of PCBP1 and C-rich 30-nucleotide-long fragments of androgen receptor mRNA [15]. The KH1 and KH3 domains bind to RNA with the corresponding Kds of 3.6 μM and 3.5 μM, while no binding has been observed for KH2. Recently, the stability of complexes formed by PCBP2 deprived of the KH3 domain versus full-length PCBP2 with the apical region of stem-loop IV of poliovirus RNA has been determined [35]. The corresponding Kds of 2.3 μM and 250 nM have been observed, showing a 10-fold lower binding affinity for the truncated protein variant. Taking the earlier published data into account, our results showed that it is not a single KH domain but at least two KH domains of the protein (probably KH 1 and KH3 domains) that are involved in the binding of PCBP2 to P1-Δ40p53 and P0-Δ40p53 RNAs.

### 2.2. Binding of PCBP2 to Selected Structural Variants of the 5′-Terminal Region of p53 mRNA

**Figure 3:**
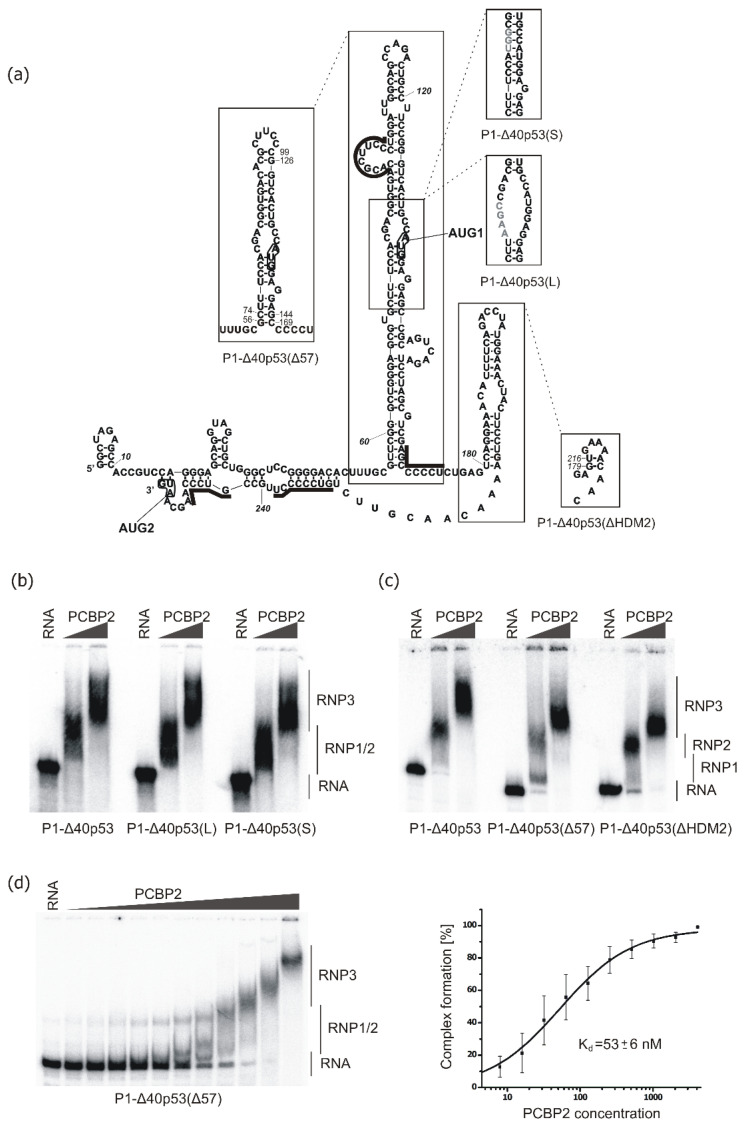
Binding of PCBP2 to the structural variants of the 5′-terminal region of p53 mRNA in vitro. (**a**) Structural variants of P1-Δ40p53 RNA: P1-Δ40p53(L)—with an internal loop in which AUG1 is located, P1-Δ40p53(S)—with a base-paired region in which AUG1 in embedded, P1-Δ40p53(Δ57) —with shortened hairpin domain and P1-Δ40p53(ΔHDM2)—with excised HDM2-binding hairpin. (**b**,**c**) EMSA assays for the constructs: P1-Δ40p53, P1-Δ40p53(L), P1-Δ40p53(S), P1-Δ40p53(Δ57) and P1-Δ40p53(ΔHDM2). The EMSA was performed by incubating [32P]-labelled RNAs with two concentrations of PCBP2: 1000 nM and 2500 nM at 4 °C for 25 min. The samples were separated on native 4% polyacrylamide gels. The first lane in each EMSA panel represents the RNA oligomer incubated without the protein. Unbound RNAs and RNP complexes are indicated. (**d**) Analysis of the complex formation of P1-Δ40p53(Δ57) with increased concentrations of PCBP2 (left panel; concentrations of PCBP2 were the same as specified in the legend to Figure 2). Graphs representing dissociation constant (Kd) curves of PCBP2 binding to P1-Δ40p53(Δ57) RNA, measured from three independent experiments, are also shown (right panel).

Subsequently, we analysed the ability of PCBP2 to form complexes with structural variants of the 5′-terminal region of p53 mRNA (Figure 3a). We have previously shown that changes in the structure of the 5′ terminus of p53 mRNA strongly influence the efficiency and speed of the translation process [30]. To find out whether structural alterations within the 5′-terminal region of p53 mRNA might affect the binding of PCBP2, PCBP2 recombinant protein was incubated with radiolabelled RNA constructs and then, EMSA was performed (Figure 3b–d). For RNAs P1-Δ40p53(L) and P1-Δ40p53(S) in which AUG1 codon is placed in a single- or double-stranded region, respectively, we observed no changes in the complex formation with PCBP2 (Figure 3b). Similarly, the removal of the small hairpin U180-A218 in P1-Δ40p53(ΔHDM2) RNA resulted in slight alterations in the formation of a complex with PCBP2 (Figure 3a,c). Thus, as predicted, the hairpin U180-A218 did not seem to be involved in PCBP2 binding. However, changes in the hairpin G56-C169, which resulted in shortening of this domain, in P1-Δ40p53(Δ57) RNA, caused a decrease in PCBP2’s ability to bind to this RNA (Figure 3a,c). This was manifested by the formation of an extra intermediate complex, RNP1, when a lower concentration of the protein was used (Figure 3c). Thus, the removal of the bottom part and the apical loop of the hairpin G56-C169 led to the partial rearrangement of the hairpin stem and changes in the pattern of the formed RNA-protein complexes (Figure 3a). 

The potential binding site of PCBP2 previously located in the loop at the 5′ side of the stem, in variant P1-Δ40p53(Δ57), is placed almost entirely in the newly-formed apical loop [30]. Additionally, the potential PCBP2 binding site at the bottom of the hairpin at the 3′ side is preserved, but the base pairing is slightly rearranged and C74-G144 is formed instead of U57-G168. To quantitatively evaluate the interactions of PCBP2 with P1-Δ40p53(Δ57) RNA, EMSA was performed with increasing concentrations of the protein (Figure 3d, left panel, and Figure S2c). Interestingly, we observed that with an increasing concentration of the protein, additional sub-complexes were formed (Figure 3d, left panel). The observed Kd value of 53 ± 6 nM was slightly lower than those determined for both P1-Δ40p53 and P0-Δ40p53 RNAs (Figure 3d, right panel). This shows the importance of the G56-C169 hairpin structure and the immediately adjacent regions in PCBP2 binding. Since a potential PCBP2 binding site seems to be present in the internal loop region between C90 and U100 we decided to analyse the interaction of PCBP2 with the isolated hairpin G56-C169 (Figure 3a). Essentially, no complex formation was observed in the range of protein concentration between 4 and 4000 nM which was applied for the earlier tested RNA constructs (Figure S2d, left panel). At a higher (5 μM and 10 μM) concentration of PCBP2, we observed the disappearance of free RNA and shifted bands on the gel, which, however, were smeary indicating that, despite only one recognition site, PCBP2 may bind to the RNA as a single molecule or as a multi-protein complex (Figure S2d, right panel). As this binding affinity seems to be characterised by Kd higher than 4 μM, presumably, only a single KH domain of PBCP2 takes part in the protein-RNA interaction of PCBP2 with the isolated hairpin G56-C169.

### 2.3. Revealing PCBP2 Binding Sites within the 5′-Terminal Region of p53 mRNA by Structural Probing 

To define the sites of the PCBP2 interactions with the 5′-terminal region of p53 mRNA we applied the Pb2+-induced cleavage method. The Pb2+-ions cleave RNA within single-stranded regions, loops and bulges, while cleavages in double-stranded regions or RNA fragments involved in the proteins’ interactions are less frequent or absent depending on steric and chemical constraints [36,37]. Recently, this approach together with the SHAPE method has allowed us to characterise the structural elements present within human and mouse 5′-terminal regions of p53 mRNAs [38,39,40].

**Figure 4:**
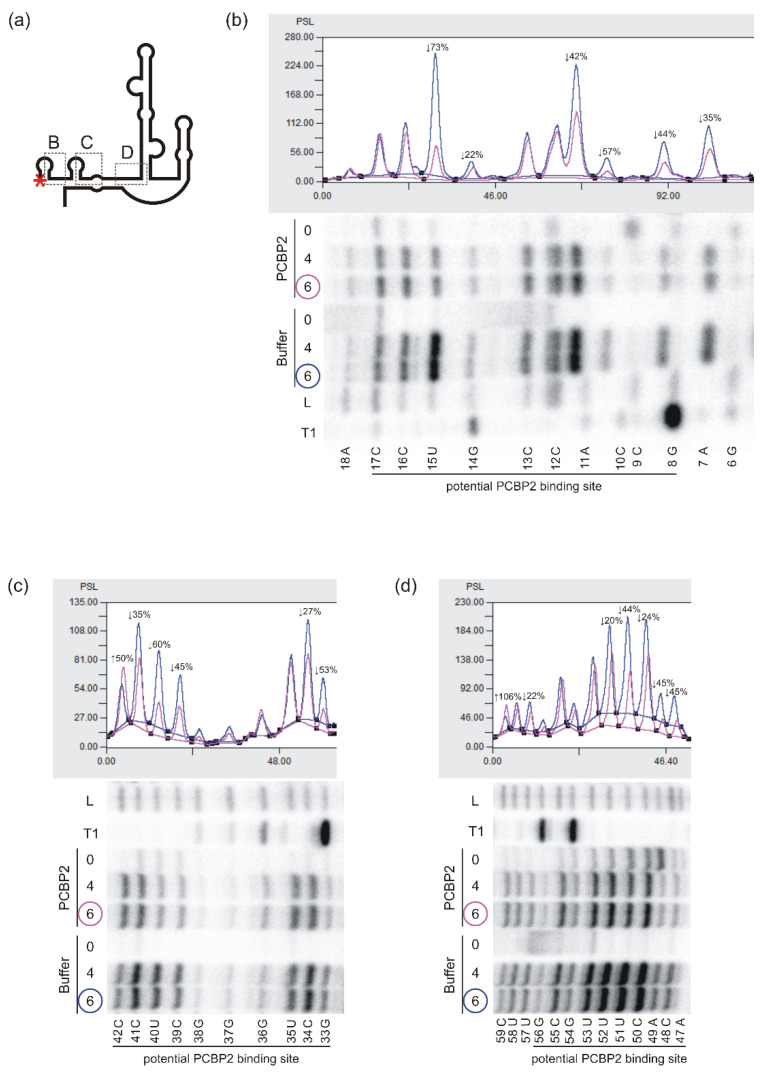
Structural probing of the P1-Δ40p53 RNA in vitro in the presence of PCBP2 by Pb2+-induced cleavage method. (**a**) Schematic representation of the structure of the P1-Δ40p53 RNA. The red asterisk denotes the 5′-[32P]-end-labelling of RNA. The analysed regions of RNA are marked with dotted-line boxes named B, C and D. (**b**–**d**) Bottom panels: the autoradiograms show the products of Pb2+-ions induced cleavage reactions analysed on 12% polyacrylamide gels in denaturing conditions. The reactions were carried out at 37 °C with 4 and 6 mM Pb2+ ions for 3 min in the presence of PCBP2 or the buffer. Lanes: L, formamide ladder; T1, limited digestion by RNase T1 in denaturing conditions. Nucleotide residues are marked on the left side of each autoradiogram. Black lines along the gels indicate potential PCBP2 binding sites. Upper panels: normalised reactivities of Pb2+-induced cleavage (6 mM Pb2+ concentration) as a function of nucleotide position.

Radiolabelled P1-Δ40p53 RNA was incubated with or without recombinant PCBP2 at a final protein concentration of 2 μM and then, the RNA was subjected to the Pb2+-induced cleavage (Figure 4 and Figure S3). We observed a weaker cleavage pattern at the 5′-proximal part of P1-Δ40p53 RNA, in the G6-C17 region, in the presence of PCBP2. Particularly, the intensity of cleavages taking place between nucleotides A11-C12 and U15-C16 decreased by approximately 40% and 70%, respectively (Figure 4b and Figure S3b). In the G6-C17 region, three two-nucleotide cytosine tracks, C9-C10, C12-C13 and C16-C17, are present (Figure 1 and Figure 4b). The nucleotide composition and the diminished Pb2+-induced cleavages in this region may indicate that it binds to PCBP2. The next region, G33-C42, also seems to be recognised by this protein since we observed much less intense cleavages, by around 30–60%, at G33, C34, C39, U40 and C41 in the presence of PCBP2 (Figure 4c and Figure S3c). At the same time, an increase in Pb2+-induced cleavage was detected at C42. Changes in the cleavage pattern were also observed in the region A47-G56, in which a three-nucleotides uridine track is present (Figure 4d and Figure S3d). Since PCBP2 has also been shown to recognise poly(U) stretch we assumed that the observed decrease in cleavage intensities in the region spanning C48 and U53 resulted from the PCBP2 binding. Thus, G6-G56, the 5′-proximal region of P1-Δ40p53 RNA, seems to be recognised and bound by PCBP2 (Figure 4 and Figure S3) despite no predicted PCBP2 binding sites in this part of the 5′ terminus of p53 mRNA (Figure 1). 

**Figure 5:**
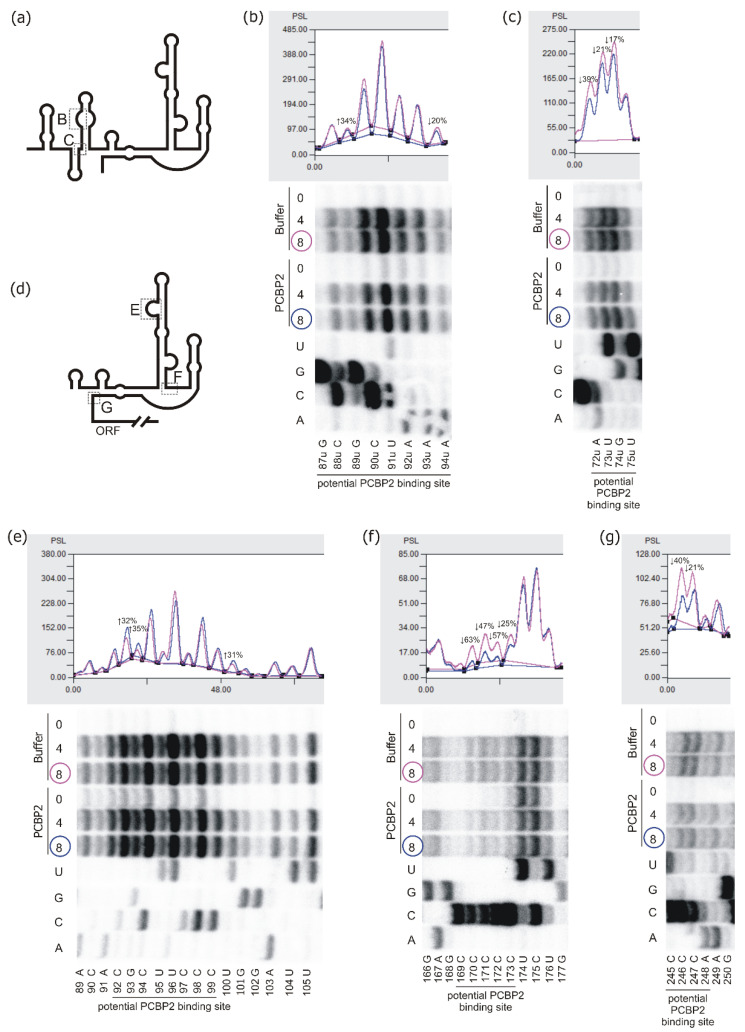
Structural analysis of the P0-Δ40p53 RNA and p53-554 RNA in the presence of PCBP2 using Pb2+-induced cleavage method. Schematic representation of the secondary structure model of P0-Δ40p53 RNA (**a**) and p53-554 RNA (**d**). The analysed regions to which PCBP2 binds are marked with dotted-line boxes B, C, E, F and G. (**b**,**c**,**e**–**g**) bottom panels: the autoradiograms show the products of Pb2+-induced cleavage reaction identified by reverse transcription method with 5′-end-[32P]-labelled DNA primers. Pb2+-induced cleavage reaction was conducted in the presence of PCBP2 protein or the buffer, at 37 °C for 3 min with Pb2+ ion concentration of 4 mM and 8 mM. Reaction products were separated on 12% polyacrylamide denaturing gels. Sequencing lines are marked A, C, G and U, respectively. Selected nucleotide residues are indicated on the left side of each autoradiogram. Black lines along the gels indicate potential PCBP2 binding sites. Upper panels: normalised reactivities for Pb2+-induced cleavage at Pb2+ ions concentration of 8 mM as a function of nucleotide position.

To analyse PCBP2 binding sites within the region extending into the 5′ direction of P0-Δ40p53 RNA, detection of Pb2+-induced cleavages by primer extension method was applied (Figure 5a–c and Figure S4a–c,h,i). A binding site for PCBP2 was predicted in the P0-P1 region, which is present at the 3′ side of a small hairpin, U49u-A72u (Figure 1). Structure probing revealed changes in the intensity of Pb2+ cleavages close to the potential PCBP2 binding site, in the region A72u-G74u. Changes were also detected in an internal loop of the hairpin G74u-C12 (Figure 5b,c and Figure S4b,c).

Appling the p53-554 RNA construct we further analysed potential PCBP2 binding sites within the large domain G56-C169. We observed changes in the intensity of Pb2+ cleavages in the C90-C99 loop and the adjacent region (Figure 5e and Figure S4e). Particularly, the U95-U100 region with three cytosine residues, C97-C99, was cleaved weaker by approximately 30% in the presence of PCBP2. It was nicely visible when a lower Pb2+ concentration was applied (Figure S4e,j). On the other hand, the 3′ side of the loop seemed to be much more accessible to Pb2+-induced cleavage with the protein present in the reaction. These results nicely confirmed the interaction of PCBP2 with the 5′ side-loop of the G56-C169 domain. 

The next binding site of PCBP2 was predicted at the bottom of the G56-C169 domain at its 3′ side and in the adjacent region where tracks of all the five cytosine residues were present (Figure 1). Decreased intensity of Pb2+ cleavages (by around 25–60%) was observed in the presence of PCBP2 in the C170-C173 region of p53-554 RNA (Figure 5f and Figure S4f,k). These changes clearly reflect the interaction of PCBP2 with this region. 

We also analysed the predicted PCBP2 binding sites located within the region between two initiation codons AUG1 and AUG2, which is involved in the proper folding of the 5′ terminus of p53 mRNA (Figure 1 and Figure 5g). In the presence of PCBP2, reduced Pb2+ cleavages were observed in the region C245-A249 of p53-554 RNA (Figure 5g and Figure S4g,l). Particularly, there was a decrease in cleavage intensity in the three cytosines track suggesting that PCBP2 was bound to this mRNA region. However, the lack of cleavages in the G231-C237 region with several cytosine residues, likely due to their involvement in the stem formation, made it impossible to map a plausible PCBP2 binding site within this region (Figure 1 and Figure S4l).

### 2.4. Major PCBP2 Binding Site Revealed by Small Angle X-ray Scattering (SAXS) 

We applied the Small Angle X-ray Scattering (SAXS) method to elucidate the PCBP2—P1-Δ40p53 RNA complex formation. SAXS is a powerful technique to study macromolecules in solution. It has been successfully used in structural studies of proteins [41], protein complexes [42], nucleic acids [43] and flexible systems like disordered proteins [44]. 

**Figure 6:**
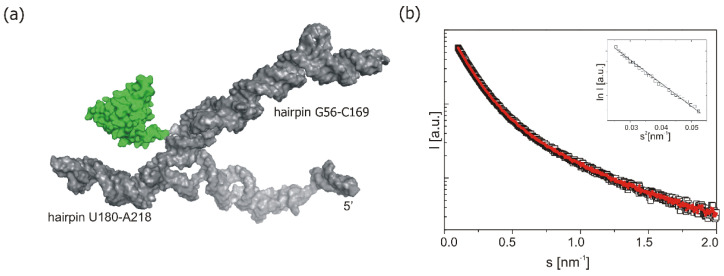
SAXS data for P1-Δ40p53 RNA—PCBP2 complex. (**a**) Quaternary structure of the complex obtained by SASREF (grey—RNA model, green—protein model). (**b**) Experimental SAXS curve used to model the quaternary structure of the P1-Δ40p53 RNA—PCBP2 complex (black line) in the region 0.01–2 nm^−1^, the red line shows the theoretical fit to the experimental data (χ^2^ = 1.34). Guinier plot shows that the analysed system is monodisperse (insert).

The SAXS analysis of the PCBP2—P1-Δ40p53 RNA complex was performed at an equimolar 9.6 μM concentration of protein and RNA components under experimental conditions comparable to those which were used in EMSA experiments. The modelling of the quaternary structure model of the complex was done using the SASREF program. The structural model is presented in Figure 6 and corresponding structural data are shown in Table 2. Analysis of structural parameters, such as the volume and the molecular weight obtained from the SAXS analysis, nicely confirmed the formation of the PCBP2—P1-Δ40p53 RNA complex with the ratio of 1:1. The 3D model clearly showed that PCBP2 protein binds to the region close to the bottom part of the 3′ side of the stem region of the G56-C169 domain (Figure 6). This is a clear confirmation of the data obtained from the mapping of the PCBP2 binding sites by Pb2+-induced cleavages (Figure 5f) and the EMSA results (Figure 3c,d). In the applied conditions, upon the equimolar concentration of protein and RNA, SAXS data analysis revealed only one major PCPB2 binding site. The single-stranded region adjacent to the bottom part of the G56-C169 domain contains the tract of five cytosine residues which might explain the preference of PCBP2 to interact with this region. There is also another place at the 5′ terminus of p53 mRNA which has a five-nucleotides poly(C) track, C233-C237. However, PCBP2 binding to this region was not observed either by the Pb2+-induced cleavage method or by SAXS. The lack of this interaction can be a result of the presence of the C233-C237 tract in the double-stranded region, whereas PCBP2 binds to single-stranded poly(C) regions [45,46]. 

### 2.5. Biological Meaning of PCBP2 Interactions with the 5′-Terminal Region of p53 mRNA 

**Figure 7:**
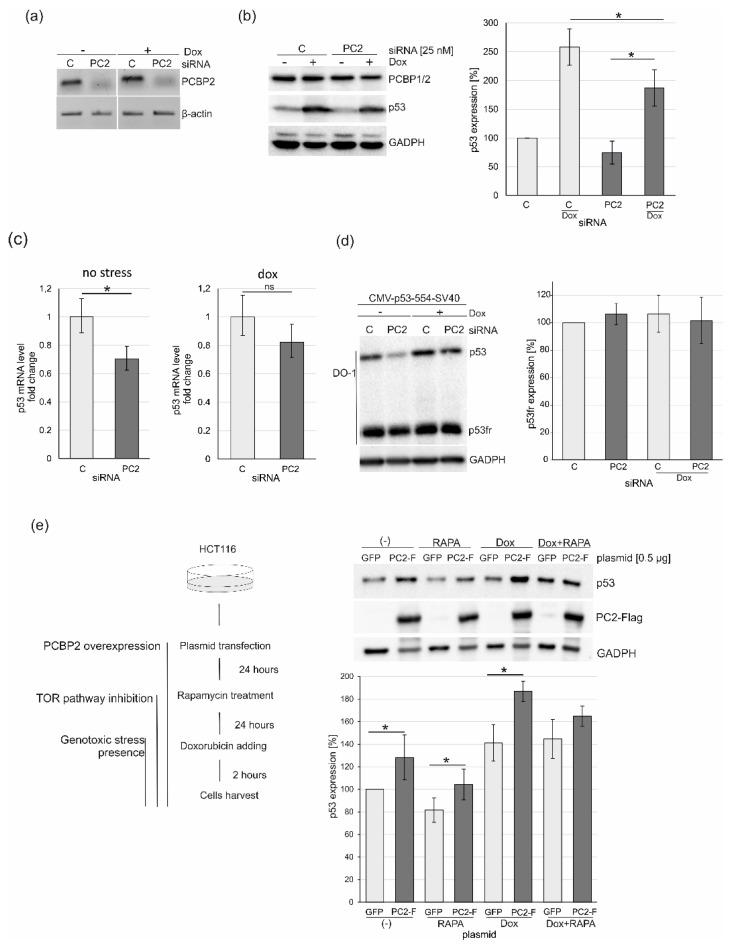
Changes in the p53 expression profile upon depletion or overexpression of PCBP2 in HCT116 cells. The cells were treated with specific PCBP2 or control siRNAs at the final concentration of 25 nM. Twenty-four hours after transfection the cells were exposed to doxorubicin (Dox, 0.5 μg/mL) for 24 h and then harvested. All experiments were repeated at least three times. *—*p*-value < 0.05, calculated using Student’s *t*-test. (**a**) Downregulation of PCBP2 by siRNA was detected by RT-PCR. (**b**) The levels of p53, PCBP1/2 and GAPDH were determined by western blots. PCBP2 was detected using an antibody (hnRNP E1/E2) that recognised both isoforms PCBP1 and PCBP2. DO-1 antibody was applied to monitor p53 protein. The bar chart shows the p53 expression level under no stress and stress conditions (Dox) in the presence of a normal or reduced level of PCBP2. (**c**) Changes in the level of p53 mRNA under PCBP2 depletion. Expression of the TP53 gene at RNA level under no stress and stress conditions was quantified as described in the Materials and Methods section. (**d**) The C-truncated p53 protein (p53fr) expressed from a vector is not affected by PCBP2 downregulation. The cells were co-transfected by specific PCBP2 or control siRNAs at the final concentration of 25 nM together with 0.5 μg of a vector (CMV-p53-554-SV40). Twenty-four hours after transfection, doxorubicin (Dox, 0.1 μg/mL) was added for 24 h and then the cells were harvested. The bar chart shows p53fr protein level under normal and stress conditions in the presence of a normal or reduced level of PCBP2 protein (right panel). (**e**) The impact of the PCBP2 overexpression on the p53 protein level under stress conditions. The cells were transfected by a vector encoded PCBP2 (PC2-F) or GFP (GFP) as a control. Schematic representation of an experiment in which the cells were treated with doxorubicin and rapamycin in the presence of overexpressed PCBP2 (left panel). The levels of p53, PC2-Flag and GAPDH were determined by western blots. The bar chart shows the p53 expression level under no stress and stress conditions (Dox, RAPA, Dox+RAPA) in the presence of normal (GFP) and overexpressed level of PCBP2 (PC2-Flag) (right panel). All experiments were repeated at least three times. *—*p*-value < 0.05, calculated using Student’s *t*-test.

Since we observed that PCBP2 can directly bind to the 5′ terminus of p53 mRNA, we addressed the biological function of these interactions. Firstly, PCBP2 was downregulated by applying specific siRNAs in HCT116 and HepG2 cells (Figure 7a,b and Figure S5). Twenty-four hours after transfection doxorubicin was added to generate genotoxic stress. A decreased level of PCBP2 mRNA was confirmed by RT-PCR (Figure 7a and Figure S5a). We observed that the downregulation of PCBP2 resulted in an approximately 20–30% lower level of the p53 protein under normal and stress conditions in HCT116 cells (Figure 7b, right panel). Similarly, PCPB2 depletion affected p53 expression at the protein level in HepG2 cells (Figure S5b). 

A decrease in the p53 protein level under PCBP2 downregulation indicated a potential involvement of PCBP2 in the translation process, particularly because we observed the direct binding of this protein to the 5′ terminus of p53 mRNA in vitro (Figure 2 and Figure 3) and complex formation in solution (Figure 6). It has earlier been demonstrated that PCBP2 is engaged in the stabilisation of several mRNAs via interactions with the 3′ ends of the transcripts [17]. Moreover, in the case of selected RNA viruses, PCBP2 has been shown to interact with their 3′ and 5′ ends leading to a loop formation that stabilises viral RNA and enhances translation [47]. As it has been suggested that PCPB2 may interact with the 3′UTR of p53 mRNA [48] we wondered whether the observed decrease in the p53 protein level upon PCBP2 depletion in the cell resulted from lower stability of p53 mRNA or disruption of the potential interactions with both 3′ and 5′ ends of p53 mRNA. Quantitative analysis revealed that under PCBP2 downregulation, changes in the PCBP2 amount affected the p53 mRNA level (Figure 7c). We observed an almost 30% lower level of p53 mRNA compared to that in the cells with the normal amount of PCBP2 (Figure 7c, left panel). It shows that PCBP2 may be involved in determining p53 mRNA stability probably via interactions with the 3′UTR of p53 mRNA. Unexpectedly, only a slight change in p53 mRNA level was observed upon PCBP2 downregulation in the presence of doxorubicin (Figure 7c, right panel). As transcription of p53 mRNA is specifically enhanced under stress conditions [49], such stress-dependent regulation is likely compensation for lower stability of p53 mRNA resulting from the downregulation of PCBP2. It also indicates that upon stress conditions, translational regulation based on PCBP2 interactions with the 5′ terminus of p53 mRNA might be involved.

To gain more details about PCBP2 engagement in the p53 expression we decided to apply p53-554 RNA, a truncated p53 mRNA transcript deprived of the 3′UTR [38]. The transcript, 554 nucleotides in length, only has a poly(A) tail added as a consequence of the SV40 late polyadenylation signal present within the vector and includes a part of the p53 coding sequence. The encoded protein, p53fr, contains the first 140 amino acids from the N-end. The cells were co-transfected with specific PCBP2 or control siRNAs together with a vector (CMV-p53-554-SV40) expressing p53fr protein. Twenty-four hours after transfection, the cells were treated with doxorubicin for the next 24 h. Both proteins, endogenous p53 and truncated p53fr, were determined by the same antibody, DO-1 (Figure 7d, left panel). As we expected, the level of the full-length p53 protein decreased upon PCBP2 downregulation compared to the control cells under stress and no stress conditions (Figure 7d, left panel). However, no changes were observed in the amount of p53fr which was translated from p53-554 RNA deprived of the 3′UTR (Figure 7d). Presumably, the lack of the 3′UTR resulted in a lower stability of truncated p53 mRNA in the cell. As a consequence, a similar amount of p53fr was detected in the presence of a normal and lower level of PCBP2 (Figure 7d). This indicates that the 3′UTR of p53 mRNA is one of the major determinants of translation regulation in which PCBP2 is involved. Since we observed a direct interaction between PCBP2 and the 5′-terminal region of p53 and the involvement of this protein in p53 mRNA stability most probably via the 3′UTR we could envisage that PCBP2 binds to both ends of p53 mRNA to contribute to the regulation of the p53 expression. It might resemble a mechanism observed in viruses in which PCBP2 interacts with the 3′UTR and with IRES within the 5′UTR to stabilise RNA and then enhance translation of viral proteins [13,47].

To further investigate the potential scenario described above HCT116 cells were transfected with plasmid vectors encoding PCBP2 or GFP and after twenty-four hours the cells were subjected to rapamycin treatment, and subsequently, two hours before cell harvest, doxorubicin was added (Figure 7e, left panel). It has previously been demonstrated that rapamycin, a TOR pathway inhibitor, impairs cap-dependent translation via inhibition of the phosphorylation of eIF4E-binding protein 1 and cap-independent mechanism or/and IRES activity is favoured [50]. We observed that overexpression of PCBP2 resulted in an approximately 20–30% higher level of the p53 amount under no stress conditions (Figure 7e, right panel). As expected, twenty-four-hour treatment with rapamycin caused a reduction of the p53 level by approx. 15% [51]. However, in the presence of overexpressed PCBP2, the level of p53 was elevated by around 10–20% (Figure 7e, right panel). PCBP2 seems likely to enhance cap-independent translation via interaction with the 5′-terminal region of p53 mRNA when translation based on the cap-dependent mode is disturbed by rapamycin. A similar effect was observed when the cells were treated with rapamycin and then with doxorubicin, however, the effect was less pronounced. It is worth noting that a strong enhancement of approx. 40% of the p53 protein level occurred in PCBP2-overexpressed cells in the presence of doxorubicin (Figure 7e, right panel). It emerges that in addition to p53 mRNA stabilisation (Figure 7c), PCBP2 is also involved in the augmentation of p53 translation via interaction with the 5′-terminal region of p53 mRNA (Figure 7e, right panel). Particularly, an impact of PCBP2 on the p53 expression is observed under stress conditions when cap-independent translation is impaired (Figure 7b,d,e).

### 2.6. Is PCBP2 a Potential New ITAF? 

We wondered whether PCBP2 might be classified as an ITAF (IRES Trans Acting Factor) for p53 mRNA. It has been shown that ITAFs are mostly nuclear or shuttle proteins that link RNA transcription/stabilisation with the translation process [52]. Despite wide research on ITAFs’ functions, their precise mode of action remains unclear [53]. It has been postulated that ITAFs might facilitate translation acting as chaperones to rearrange the RNA structure or to allow other proteins to interact with the ribosomal complex [54]. In the case of p53 mRNA, only a few proteins have been identified so far which can bind to the 5′-terminal region of p53 mRNA and some of them have been characterised or categorised as ITAFs [5,9]. They include, among others, annexin A2, PTB and PSF/SFPQ (PTB associated Splicing Factor) which have been shown to bind and positively regulate p53 IRES activity [55,56]. DAP5 (eIF-4G homolog, Death Associated Protein 5) has also been proposed as an ITAF since it enhances translation of p53 and its isoform, Δ40p53 [57]. More recently, a model has been suggested based on the interactions of the 5′ terminus of p53 mRNA with two proteins: TC80 (Translational Control Protein 80) and RHA (RNA Helicase A), which are additionally able to bind each other to eventually increase IRES activity upon stress conditions [58].

Another model of regulation of p53 protein synthesis through binding to the 5′UTR is based on the competition between ribosomal protein L26 (RPL26) and nucleolin [59,60,61]. It has been shown that RPL26 enhances p53 translation after DNA damage which depends upon interactions between the 5′ and 3′ untranslated regions of p53 mRNA. On the other hand, nucleolin suppresses the translation and both proteins interact with the same 5′-3′ UTR interacting region. Moreover, nucleolin can oligomerise stabilising the double-strand RNA structure. RPL26 can disrupt nucleolin dimerisation reducing the interaction between both proteins and attenuating p53 induction by RPL26 [59,60,61]. The authors postulate a model in which the base pairings between the 5′ and 3′ untranslated regions of p53 mRNA are critical for both translational repression and stress induction of p53 by nucleolin and RPL26 [59]. 

Interestingly, we noticed a similarity in the sequence composition and the structural location of PCBP2 potential binding site between C90 and U100 in the 5′-terminal region of p53 mRNA (Figure 1) and those characterised within domains IV/C in the 5′-terminal region of Coxsackievirus B3 RNA and Poliovirus I [35]. These recognition sites are placed within the upper part of the biggest structural element of these 5′ termini of RNAs. Interactions of PCBP2 with these specific binding sites might be important for harbouring other translational factors or facilitating the placing of the ribosome in the correct position [35]. It is worthy of note that in the 5′-terminal region of p53 mRNA, the region of possible interaction with PCBP2 lies in the centre of the sequence 5′GACGGUGACACGCUUCCCU3′ which has been proposed to base-pair with the 3′ untranslated region of p53 mRNA. Although this region seemed to weakly bind to PCBP2, its involvement in RNA-protein or protein-protein interactions which brings the 5′ and 3′ ends of p53 mRNA in close proximity offers an attractive possibility. 

**Figure 8:**
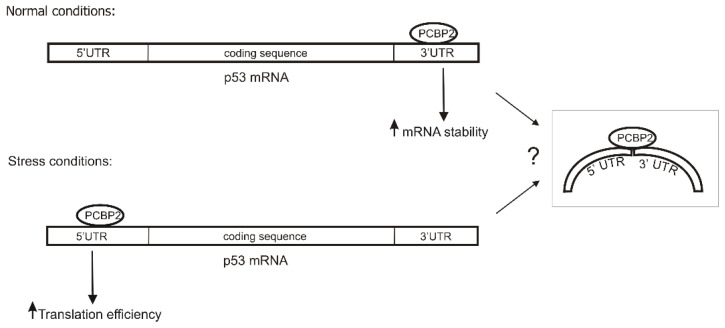
Model of the regulation of p53 expression with the involvement of PCBP2 interacting with the 5′ and 3′ ends of p53 mRNA. Under normal conditions, PCBP2 increases the stability of p53 mRNA, probably via the interactions with the 3′UTR, while in the presence of stress factors PCBP2 binds to the 5′ terminus of p53 mRNA modulating p53 translation. PCBP2 is likely to bind to both ends of p53 mRNA simultaneously under stress conditions, which resembles the interactions with viral RNAs (grey box).

Thus, in the p53 expression, PCBP2 emerges as a double-function factor (Figure 8). It is engaged in the enhancement of p53 RNA stability probably via interacting with the 3′ end of p53 mRNA, while under stress conditions, it stimulates p53 translation via specific binding to the 5′-terminal region of p53 mRNA (Figure 8). Moreover, we envisage that PCBP2 might simultaneously interact with both ends of p53 mRNA that resembles its action in the enterovirus life cycle. Now then, is PCBP2 a p53 ITAF? Considering the previously characterised functions of this protein together with our current observations we postulate that PCBP2 may play a role of an ITAF in p53 translation. Further research is, however, needed to reveal the precise mode of action of PCBP2 and to determine how it interacts with other ITAFs to build the intricate network of protein-p53 mRNA interactions which is responsible for the modulation of p53 expression. 

## 3. Materials and Methods

### 3.1. Cell Culture and Stress Induction

HCT116 cells (CCL-247™, originally obtained from ECACC) were cultured in McCoy’s medium (Sigma-Aldrich, Taufkirchen, Germany). The medium solution was supplemented with 10% foetal bovine serum (Biowest, Riverside, MO, USA), non-essential amino acids (Sigma), 100 U/mL of penicillin G (Sigma-Aldrich, Taufkirchen, Germany) and 0.1 mg/mL of streptomycin sulphate (Sigma-Aldrich, Taufkirchen, Germany). The cells were maintained at 37 °C in the atmosphere of 5% carbon dioxide. Genotoxic stress was generated by the addition of doxorubicin to a final concentration of 0.1 or 0.5 μg/mL (Sigma-Aldrich, Taufkirchen, Germany). Rapamycin was added to a final concentration of 50 nM (Sigma-Aldrich, Taufkirchen, Germany). The cells were exposed to stress conditions for 2 or 24 h, then harvested. 

### 3.2. siRNA and Plasmid Transfection

The cells between passages 4 and 20 were used for each transfection. The transfection was performed when a confluence of the cells reached 50–80%. The ON-TARGETplus SMARTpool Human PCBP2 siRNAs and the ON-TARGETplus Non-targeting SMARTpool siRNA were purchased from Dharmacon Inc, Lafayette, CO, USA. The cells were transfected by siRNAs at a final concentration of 10 or 50 nM using the RNAiMax Lipofectamine according to the manufacturer’s transfection protocol (Thermo Fisher, Walthan, MA, USA). The plasmid encoding PCBP2-Flag was purchased from OriGene Technologies Inc, Rockville, MD, USA (Cat No. RC210035). The vector (CMV-p53-554-SV40) encoding p53fr, which comprises the first 140 amino acids of full-length p53, was obtained earlier in our laboratory [38]. The cells were transfected with 0.5 μg of the vector applying Lipofectamine3000 according to the transfection protocol (Thermo Fisher, Walthan, MA, USA). The transfected cells were washed with PBS (Sigma-Aldrich, Taufkirchen, Germany), given fresh medium 24 h after the transfection and then harvested 48 h post-transfection.

### 3.3. RNA Isolation, RT-PCR and Quantitative PCR (qPCR)

For RT-PCR, total RNA was isolated from HCT116 or HepG2 cells using TriReagent according to the standard protocol. Reverse transcription was performed as previously described [62]. Briefly, the cDNA was prepared from 200 ng of RNA using 100 ng of oligo(dT)18 primer and 100 units of SuperScriptTM IV reverse transcriptase (Thermo Fisher, Walthan, MA, USA). Equal volumes of cDNA were used to amplify DNA fragments of PCPB2 and β-actin using specific primers listed in Table S1. Quantitative PCR was performed as previously described [6]. Shortly, quantitative PCRs (20 μL) were performed on aliquots of cDNA samples (5 μL, dilution 1:50) using 5× HOT FIREPol^®^ EvaGreen^®^ qPCR Mix Plus (Solis Biodyne, Tartu, Estonia) applying LightCycler 480 System (Roche, Penzberg, Germany). The cycling conditions were 12 min at 95 °C, followed by 40 cycles consisting of 15 s at 95 °C, 20 s at 60 °C and 20 s at 72 °C. All tested primers met the criteria of efficiency of 90–110% and r^2^ > 0.985. The results were expressed as a relative quantity according to the equation RQ = 2ΔΔCt ± standard deviation (SD). Differences between samples were evaluated using a two-way Student’ *t*-test.

### 3.4. Western Blot

The cells were lysed in a buffer containing: 62.5 mM Tris-HCl pH 6.8, 2% SDS, 10% glycerol, 50 mM DTT and 1× of protease inhibitor mix (Sigma-Aldrich, Taufkirchen, Germany). The total cell lysates were incubated for 5 min at 95 °C, loaded on a 10% or 15% SDS-PAGE gel and proteins were separated and then transferred onto a PVDF membrane (GE Healthcare, Chicago, IL, USA). The blot was probed with antibodies specific to p53 (DO1) (sc-126, Santa Cruz, Dallas, TX, USA), PCBP1/2 (hnRNP E1/E2; F-6, Santa Cruz, Dallas, TX, USA), FLAG (PA1-984B, Thermo Fisher, Walthan, MA, USA) and GAPDH (6C5, Sigma-Aldrich, Taufkirchen, Germany). The primary antibody was detected by goat Anti-Mouse-HRP or goat Anti-Rabbit-HRP (31460, Thermo Fisher, Walthan, MA, USA) and visualised using a chemiluminescent visualisation system (ECL) (Thermo Fisher, Walthan, MA, USA). All densitometric values were normalised to the values of GAPDH, used as internal controls (Figure 7).

### 3.5. Protein Overexpression and Purification

PCBP2 protein was overexpressed and purified as previously described for hnRNP K [6]. Briefly, *E. coli* (BL21 Star (DE3) pLysS) (C602003, Thermo Fisher, Walthan, MA, USA) bacteria were transformed with the modified pMCSG48 plasmid encoding the wild type PCBP2 protein by a heat shock method. Then, the bacteria were inoculated in 250 mL of the LB medium containing 100 μg/mL ampicillin. The cells were grown at 37 °C and when the OD600 of the culture reached 0.5–0.6, IPTG was added to a final concentration of 0.4 mM. After the induction of a fusion protein, the culture was incubated for 4 h at 30 °C. The bacteria paste was resuspended in a lysis buffer: 50 mM NaH2PO4 pH 8.0, 100 mM NaCl, 20 mM imidazole, 6% glycerol, 1 mM DTT, 1 mM PMSF and containing 0.2 mg/mL lysozyme (BioShop, Burlington, ON, Canada). After incubation on ice for 30 min, the lysate was sonicated for 5 min: each impulse of 2 s and 10 s of intervals on ice. Cell debris was removed by centrifugation at 16,000× *g* rpm for 30 min at 4 °C.

Affinity chromatography was used to purify the PCBP2 protein. After the protein binding with Ni-NTA resin (Qiagen, Germantown, MD, USA), the column was washed with the lysis buffer. Then, the protein was eluted from the Nickel column with an elution buffer: 50 mM NaH2PO4 pH 8.0, 100 mM NaCl, 500 mM imidazole, 6% glycerol, 1 mM DTT, 1 mM PMSF. To remove the N-terminal tag, a TEV protease at a final concentration of 10 U was used. After 1 h digestion at 4 °C, the buffer was replaced with lysis buffer on the CentriPure P Hydrated Gel Filtration Columns (emp BIOTECH, GmbH, Berlin, Germany) to get rid of imidazole. The eluate containing the PCBP2 protein was applied to the Ni-NTA charged column to remove the tag and any undigested protein. The flow-through was collected and the protein sample was concentrated using the Amicon Ultra-15 Centrifugal Filter Units (10 kDa cut off) according to the manufacturer’s protocol (Sigma-Aldrich, Taufkirchen, Germany). The induction of fusion protein in bacteria cells and the purification of PCBP2 were verified using SDS-PAGE in standard conditions. Gels were stained by the Coomassie brilliant blue (Figure S1).

### 3.6. RNA Transcription In Vitro and RNA Purification 

The dsDNA templates for the preparation of P0-Δ40p53, P1-Δ40p53, P1-Δ40p53(L), P1-Δ40p53(S), P1-Δ40p53(Δ57), P1-Δ40p53(ΔHDM2), p53-554 and isolated hairpin G56-C169 RNAs had been obtained previously in our laboratory [38,39,63]. For transcription in vitro, the dsDNA templates were first linearised with Csp45 or Xba1 restriction enzymes (Thermo Fisher, Walthan, MA, USA) in accordance with the manufacturer’s protocols. Transcription reactions were performed using the TranscriptAid T7 High Yield Transcription Kit (Thermo Fisher, Walthan, MA, USA) as recommended by the manufacturer’s protocol and with an addition of 4 mM guanosine. After the transcription, RNAs were purified using the GeneJet RNA Isolation Kit (Thermo Fisher, Walthan, MA, USA).

### 3.7. Electrophoretic Mobility Shift Assay (EMSA)

Prior to the formation of RNA-protein complexes, the RNA was radiolabelled at the 5′ end with *γ*-[32P] ATP and T4 polynucleotide kinase according to the standard procedure. Then, the RNA was denatured at 65 °C for 5 min in a buffer containing: 20 mM HEPES pH 8.0 and 100 mM KCl, cooled on ice and supplemented with MgCl2 to 5 mM final concentration. The RNA-protein binding reaction was conducted in a buffer containing 25 mM NaH2PO4 pH 8.0, 50 mM NaCl, 25 mM KCl, 2.5 mM MgCl2, 10 mM imidazole, 0.5 mM DTT, 0.5 mM PMSF, 7% glycerol, 0.5 U/μL RNasin and competitors: 75 ng/μL BSA and 100 ng/μL yeast tRNA for unspecific binding of proteins. Subsequently, 1000 or 2500 nM of the PCBP2 protein and the [32P]-labelled RNA were incubated at/for: 37 °C/10 min, 25 °C/15 min or 4 °C/25 min. For Kd measurement, 3.9, 7.8, 15.6, 31.3, 62.5, 125, 250, 500, 1000, 2000 and 4000 nM of the PCBP2 and the [32P]-labelled RNA was incubated at 4 °C for 25 min. Additional, higher PCBP2 concentrations of 5 and 10 μM were used for the isolated G56-C169 hairpin. Then, the samples were loaded on a native 4% polyacrylamide gel (acrylamide to bisacrylamide, 29:1, 2.5% glycerol and TB buffer. Electrophoresis was conducted at 4 °C for 2 h. The gel was dried and visualised using FLA 5100 image analyser (Fuji). For Kd calculation, the Origin 6 software (OriginLab Corporation, Northampton, MA, USA) was used.

### 3.8. Pb2+-Induced Cleavage and Primer Extension

Prior to the cleavage reaction with Pb2+ ions, RNA was denatured in the 1× Pb buffer: 40 mM NaCl, 10 mM Tris-HCl pH 7.3, 5 mM MgCl2 for 3 min at 65 °C, incubated for 5 min at 37 °C and cooled on ice. The pre-treated RNA was divided into two separate pools: one was supplemented with PCBP2 (to a final concentration of 2 μM) suspended in 1× Pb buffer and the other only with 1× Pb buffer. After incubation for 15 min on ice, each panel was divided into three samples. Lead(II) acetate solution was added to a final concentration of 4 mM and 6 or 8 mM and an equal volume of water was added to the control reaction. The samples were incubated at 37 °C for 3 min and the reactions were terminated by mixing their aliquots with the same volume of 8 M urea, 20 mM EDTA solution. The cleaved RNA was precipitated by 0.3 M sodium acetate pH 5.2 supplemented with 1 μL of glycogen (20 mg/mL) and 3 volumes of 96% ethanol. In the case of 5′-end [32P]-radiolabelled P1Δ40p53 RNA, the reaction products were resolved on 12% polyacrylamide, 7 M urea, 1× TBE gel and the gel was dried, followed by radioisotope imaging with FLA 5100 image analyser (FujiFilm, Cambridge, MA, USA). In the case of non-radiolabelled P0-Δ40p53 and p53-554 RNAs, primer extension reaction was performed with 5′-end [32P]-radiolabelled primers (Table S1) using SuperScript IV First-Strand Synthesis System (Thermo Fisher, Walthan, MA, USA) according to the manufacturer’s protocol and reaction products were resolved as described above. Band intensities of the selected lanes were analysed by Multi Gauge V3.0 software (FujiFilm, Cambridge, MA, USA).

### 3.9. Small Angle X-ray Scattering (SAXS)

Small Angle X-ray Scattering data were collected at the P12 beamline of PETRAIII storage ring at the DESY (Deutsches Electronen Synchrotron) in Hamburg, Germany. 20 μL samples of the P1-Δ40p53-PCBP2 complex at a final concentration of 9.6 μM in the buffer containing 25 mM NaH2PO4 pH 8.0, 50 mM NaCl, 25 mM KCl, 2.5 mM MgCl2, 10 mM imidazole, 5% glycerol and 1mM EDTA were analysed. All SAXS data were collected over the wave vector range of 0.0088–5 nm^−1^ and at 15 °C. ATSAS package was used for all manipulations of raw data [64]. Modelling of the quaternary structure of the P1-Δ40p53—PCBP2 complex was done using SASREF modelling software (v. 3.0.4, EMBL, SAXS Group, Hamburg, Germany) [65]. A tertiary structure model of P1-Δ40p53 RNA was generated using the RNA Composer server [66,67]. As an input, the RNA sequence and the secondary structure in the dot-bracket notation were used, based on the previously published data [29,38]. The quaternary structure of PCBP2 protein was generated using the SWISS-MODEL (Biozentrum, The Centre for Molecular Life Sciences, University of Basel, Basel, Schwitzerland) [68]. Theoretical calculations of the structural parameters (radius of gyration, volume) were performed in CRYSOL (in ATSAS package v. 3.0.4, EMBL, SAXS Group, Hamburg, Germany) [69]. 

## 4. Conclusions

We have shown that PCBP2 binds to the 5′-terminal region of p53 mRNA using oligonucleotides which correspond to two length-variants of this region, P0-Δ40p53 and P1-Δ40p53 RNAs and the electrophoretic mobility shift assays (Figure 2). The determined Kd values and earlier published data on interactions of PCBP2 with RNA targets suggest that in the binding process, at least two KH domains of the protein (and not a single one) are involved. An analysis of the mutated variants of the 5′-terminal fragment of p53 mRNA shows that the preservation of its tertiary structural features determines the proper formation of complexes with PCBP2 (Figure 3). Moreover, it seems that more than one PCBP2 molecule binds to the 5′-terminal region of p53 mRNA. Thus, PCBP2 may simultaneously bind to several sites similarly as shown for the multi-complex of PCBP2 and IRES RNA of poliovirus [70]. The important role of PCBP2 in IRES-dependent translation of viral, c-myc [71], FHL3 [72], Hr [73] mRNAs [34,35] has been documented. We hypothesise that PCBP2 is also involved in a cap-independent translation of p53 mRNA for which the entire appropriate structure of the 5′ terminus of this mRNA is required. 

Based on Pb2+ structural probing data the predicted binding sites of PCBP2 within the 5′-terminal region of p53 mRNA have been verified. PCBP2 binds to the G56-C169 hairpin domain and poly(C) track located between two initiation codons AUG1 and AUG2. Additional binding sites for PCBP2 seem to be present in the 5′-proximal part of P1-Δ40p53 RNA (Figure 4 and Figure 5). Importantly, upon equimolar concentration of the protein and RNA, SAXS data analysis reveals only one major PCPB2 binding site, in the single-stranded region adjacent to the bottom part of the G56-C169 domain (Figure 6). This region contains the tract of five cytosine residues which may explain the preference of PCBP2 to bind to this site.

The eCLIP data has shown that PCBP2 binds to the 3′UTR of p53 mRNA [48]. Our results suggest that the protein is engaged in the enhancement of p53 mRNA stability probably via interacting with the 3′ end of p53 mRNA under normal conditions (Figure 7). Under stress conditions, it stimulates p53 translation via specific binding to the 5′-terminal region of p53 mRNA, thus PCBP2 emerges as a double-function factor in the p53 expression (Figure 8).

## Figures and Tables

**Table 1:** MS data for poly(C)-binding protein 2 from representative RNA-centric affinity chromatography experiments for cell lines MCF-7, HepG2 and HT-29 and each of the tested conditions [6]. The table shows the numbers of sequence matches and sequence coverage [%] for PCBP2 in a particular experiment. Dox—doxorubicin treatment generating stress conditions.

Cell Line Type	MCF-7				HepG2				HT-29			
RNA (construct)	P1-Δ40p53		P0-Δ40p53		P1-Δ40p53		P0-Δ40p53		P1-Δ40p53		P0-Δ40p53	
Stress factor	-	Dox	-	Dox	-	Dox	-	Dox	-	Dox	-	Dox
	Poly(C) Binding protein 2 (PCBP2)											
Peptide numbers (sequence matches)	10	11	11	11	5	4	7	3	8	8	8	6
Sequence coverage	34.0	46.6	31.8	47.1	31.8	14.3	34.8	16.2	37.5	33.4	39.7	24.9

**Table 2:** SAXS data collection and structural parameters derived for complex P1-Δ40p53—PCBP2, P1-Δ40p53 RNA and PCBP2.

Sample	Complex mRNA p53-PCBP2	mRNA p53	PCBP2
**Data collection**			
Instrument	P12, PETRA III		
s range (nm^−1^)	0.0088-5.0		
Wavelength (Å)	1.24		
Temperature	15 °C		
**Structural parameters**			
I(0) (from p(r)) (arbitrary units)	0.4970	0.1472	0.4071
R_g_ (from p(r)) (nm)	6.77	6.37	4.01
I(0) (from Guinier) (arbitrary units)	0.52057	0.15571	0.3902
R_g_ (from Gunier) (nm)	6.79	6.53	3.98
R_g_ (theoretical) (nm)	6.013	6.47	3.3
D_max_ (nm)	20	19.00	12.5
Porod volume estimate (nm^3^)	9125	7352	3002
Dry volume calculated from the model (nm^3^)	8893	7112	2962
**Molecular mass determination**			
Contrast (Δρ × 10^10^ cm^−2^)	3.047		
Experimental molecular weight (Da)	120,890	81,870	38,901
Theoretical molecular weight (Da)	120,260	81,680	38,580
**Software used**			
Primary data reduction	PRIMUS		
Data processing	PRIMUS		
Quaternary structure modelling	SASREF		
Computation of model intensities	CRYSOL		
3D graphics representation	PyMOL		
Abbreviations: Rg—Radius of gyration, Dmax—maximum dimension			

## Data Availability

The data sets and materials generated during and/or analysed during the current study are available from the corresponding author on reasonable request.

## Supplementary Materials

The following are available online at https://www.mdpi.com/article/10.3390/ijms222413306/s1.
