# Supplementary material for: Poly(C)-binding Protein 2 Regulates the p53 Expression via Interactions with the 5′-Terminal Region of p53 mRNA
Source: Int J Mol Sci. 2021 Dec 10;22(24):13306. doi: 10.3390/ijms222413306 (PMC8708005; doi:10.3390/ijms222413306)
Supplement: Supplementary file 1 [file ijms-22-13306-s001.zip › ijms-1480573-supplementary.pdf]

Supplementary material

Poly(C)-binding protein 2 regulates the p53 expression via interactions with the 5'-terminal region of p53 mRNA

Damian M. Janecki, Agata Swiatkowska*, Joanna Szpotkowska, Anna Urbanowicz, Martyna Kabacińska, Kamil Szpotkowski, and Jerzy Ciesiołka*

Institute of Bioorganic Chemistry, Polish Academy of Sciences, Noskowskiego 12/14, 61-704 Poznan, Poland

* Correspondence: jerzy.ciesiolka@ibch.poznan.pl (J.C.), agaswiat@ibch.poznan.pl (A.S.)

Table S1: Primers applied in RT-PCR and qPCR

Primers		
**PCBP2**	Forward	5'-CACCAGTTGGCAATGCAACAGTCT-3'
	Reverse	5'-ATGCATCCAAACCTGCCCAATAGC-3'
**ß-actin**	Forward	5'-AGAGCAAGAGAGGCATCCTG-3'
	Reverse	5'-CGACGTAGCACAGCTTCTCC-3'
**P53**	Forward	5'-CAGATCCTAGCGTCGAGCCCC-3'
	Reverse	5'-CTGGGTCTTCAGTGAACCATTGTTC-3'
**HPRT**	Forward	5'-TGACCTTGATTTATTTTGCATACC-3'
	Reverse	5'-CGAGCAAGACGTTCAGTCCT-3'
**No.1**	Forward	5′-GGCTCTAGACTTTTGAGAAGC-3′
**No.2**	Forward	5′-CCCAGCCCGAACGCAAAG-3′
**No.3**	Forward	5′-GGCTCCTCCATGGCAGTG-3′
**No.4**	Forward	5′-CGTTGTTTTCAGGAAGTAGTTTCC-3′
**No.5**	Forward	5′-CAGTGAACCATTGTTCAATATCG-3′

Supplementary Figures' description

Figure S1 Overexpression and purification of a fusion protein PCBP2. Transformed *E. coli* (BL21 Star (DE3) pLysS) bacteria with the modified pMCSG48 plasmid encoding PCBP2 were cultured in LB medium containing ampicillin and were induced by IPTG. The bacteria were lysed by lysozyme and sonicated. After centrifugation the supernatant containing a fusion protein was used for purification of PCBP2 by nickel affinity chromatography.

(a and b) IPTG induction of a fusion protein in bacteria and all steps of PCBP2 purification analysed by SDS-PAGE and the Coomassie brilliant blue staining

Figure S2 Two repetitions of EMSA experiments used for calculation of dissociation constants (Kds) of PCBP2 binding to P1-Δ40p53 (a), P0-Δ40p53 (b), P1-Δ40p53(Δ57) (c) RNAs and the isolated G56-C169 hairpin (d). EMSA assays were performed by incubation of the [^{32}P]-labelled RNAs with increased concentrations of PCBP2 3.9, 7.8, 15.6, 31.3, 62.5, 125, 250, 500, 1000, 2000, 4000 nM at 4°C for 25 minutes. In the case of the isolated hairpin G56-C169 (panel d, on the right) additional, higher PCBP2 concentrations of 2.5, 5 and 10 μM were used. The samples were separated on native 4% polyacrylamide gels. The first lane in each EMSA panel represents the RNA oligomer incubated without the protein. Unbound RNAs and RNP complexes are indicated

Figure S3 Structural probing of the 5'-terminal region of P1-Δ40p53 mRNA in the presence of PCBP2 by Pb^{2+}-induced cleavage. (a) Schematic representation of the structure of P1-Δ40p53 RNA. Red asterisk denotes the 5′-[^{32}P]-end-labelling of RNA. The analysed regions of PCBP2 binding are marked by dotted-line boxes named B, C and D, respectively. **(b-d)** Bottom panels: the autoradiograms show the products of Pb^{2+}-ions induced cleavage reactions analysed on 12% polyacrylamide gels in denaturing conditions. The reactions were carried out at 37°C with 4 and 6 mM Pb^{2+} ions for 3 min in the presence PCBP2 or the buffer. Lanes: L, formamide ladder; T1, limited digestion by RNase T1 in denaturing conditions. Selected nucleotide residues are marked on the left side of each autoradiogram. Black lines along the gels indicate potential PCBP2 binding sites. Upper panels: normalized reactivities of Pb^{2+}-induced cleavage (4 mM Pb^{2+} final concentration) as a function of nucleotide position. **(e)** Autoradiograms show the products of Pb^{2+}- induced cleavage reaction of the P1-Δ40p53 RNA. Selected guanine residues are marked on the left side of each autoradiogram. Dotted-line boxes indicate fragments shown in Figures 4b-d and S3 b-d

Figure S4 Structure probing of the 5'-terminal region of P0-Δ40p53 and p53-554 mRNA with and without PCBP2. Schematic representation of the secondary structure model of the P0-Δ40p53 RNA **(a)** and p53-554 RNA **(d).** The analysed regions of PCBP2 binding are marked by dotted-line boxes named B, C, E, F, and G. **(b, c, e, f, g).** Bottom panels: autoradiograms representing the products of Pb^{2+}-induced cleavage identified by reverse transcription reaction with 5′-end-[^{32}P]-labelled DNA primers. Pb^{2+}-induced cleavage reaction was conducted in the presence of PCBP2 protein or the buffer, at 37°C for 3 minutes with 4 mM and 8 mM Pb^{2+} ions. Reaction products were separated on 12% polyacrylamide denaturing gels. Sequencing lines A, C, G and U are indicated, respectively. Selected nucleotide residues are marked on the left side of each autoradiogram. Black lines along the gels indicate potential PCBP2 binding sites. Upper panels: normalized reactivity of Pb^{2+}-induced cleavage at 4 mM concentration of Pb^{2+} ions as a function of nucleotide position. **(h-l)** schematic representation of the secondary structure model of the 5' terminal region of P0-Δ40p53 RNA (**h-i**) and p53-554 RNA **(j-l)** with the primer binding site marked by red arrow and corresponding autoradiograms showing the products of Pb^{2+}-induced cleavage identified by reverse transcription reaction with 5′-end-^{32}P-labeled DNA primers. Dotted-line boxes indicate fragments shown in Figures 5 b, c, e, f, g and S4 b, c, e, f, g

Figure S5 Decrease of p53 protein expression under depletion of PCBP2 in HepG2 cells. (a) A decrease in the PCBP2 mRNA level was detected by RT-PCR. The cells were treated with specific PCBP2 or control siRNAs at the final concentration of 50 nM. Twenty-four hours after transfection the cells were exposed to doxorubicin (Dox, 0.5 μg/mL) for 24 hours and then harvested. PC2 - specific siRNA against PCBP2 mRNA, C - control siRNA. β-actin was used as a control. **(b)** The level of PCBP1/2, p53 and GAPDH was determined by western blots. PCBP2 was detected by using antibody (hnRNP E1/E2) against both isoforms PCBP1 and PCBP2

Figure S1

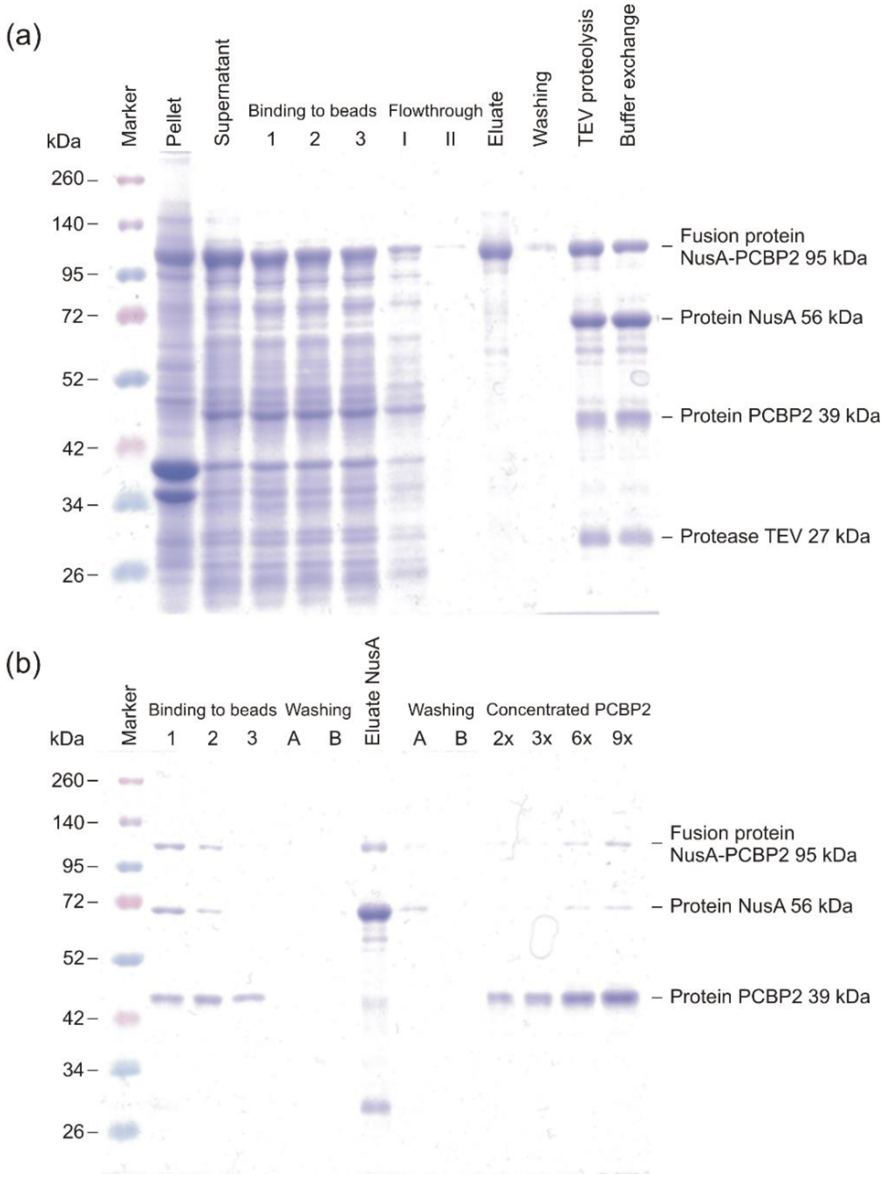

Figure S2

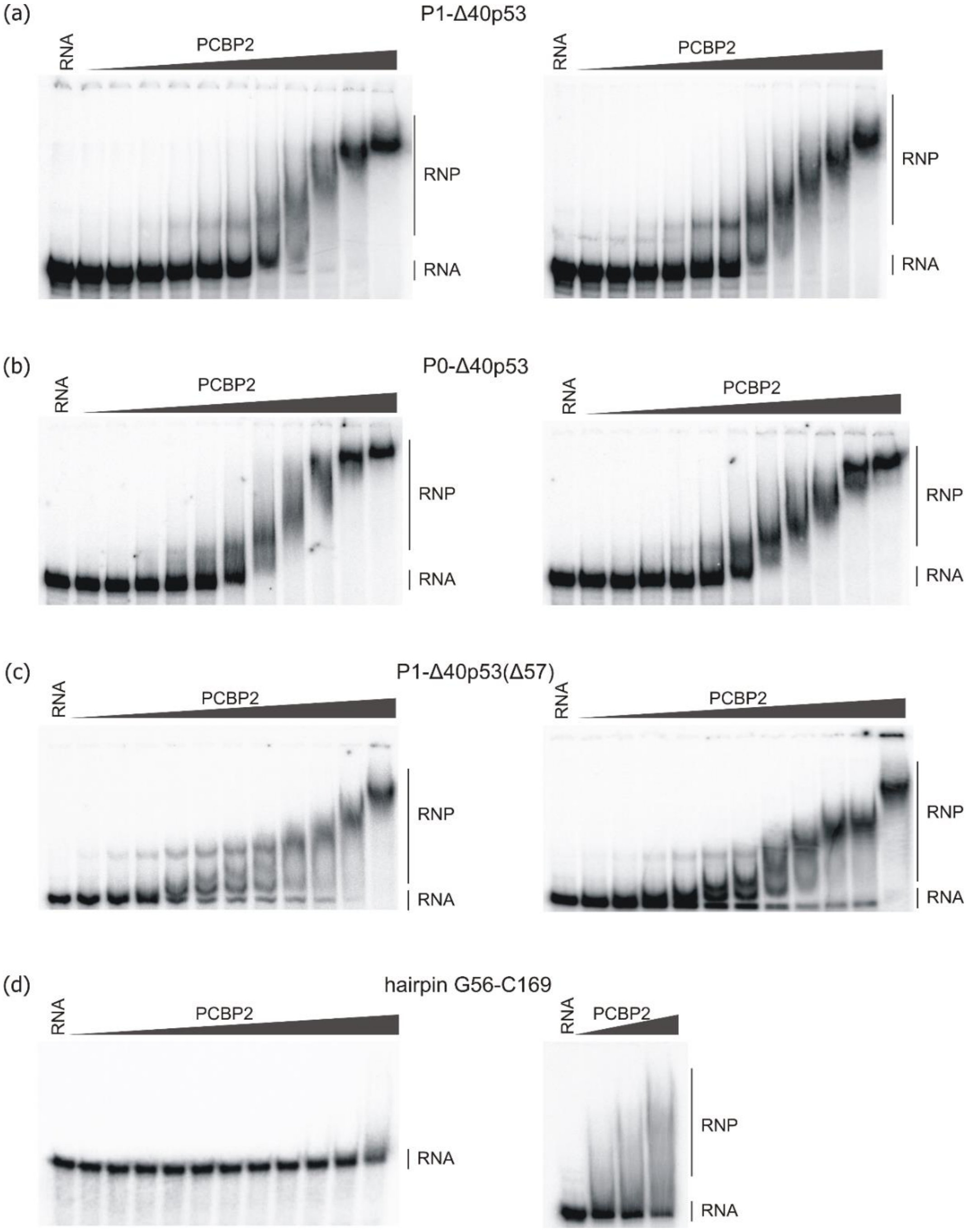

(a)
P1-Δ40p53
RNA
PCBP2
RNP
RNA
RNA
PCBP2
RNP
RNA
(b)
P0-Δ40p53
RNA
PCBP2
RNP
RNA
RNA
PCBP2
RNP
RNA
(c)
P1-Δ40p53(Δ57)
RNA
PCBP2
RNP
RNA
RNA
PCBP2
RNP
RNA
(d)
hairpin G56-C169
RNA
PCBP2
RNA
RNA
PCBP2
RNP
RNA

Figure S3

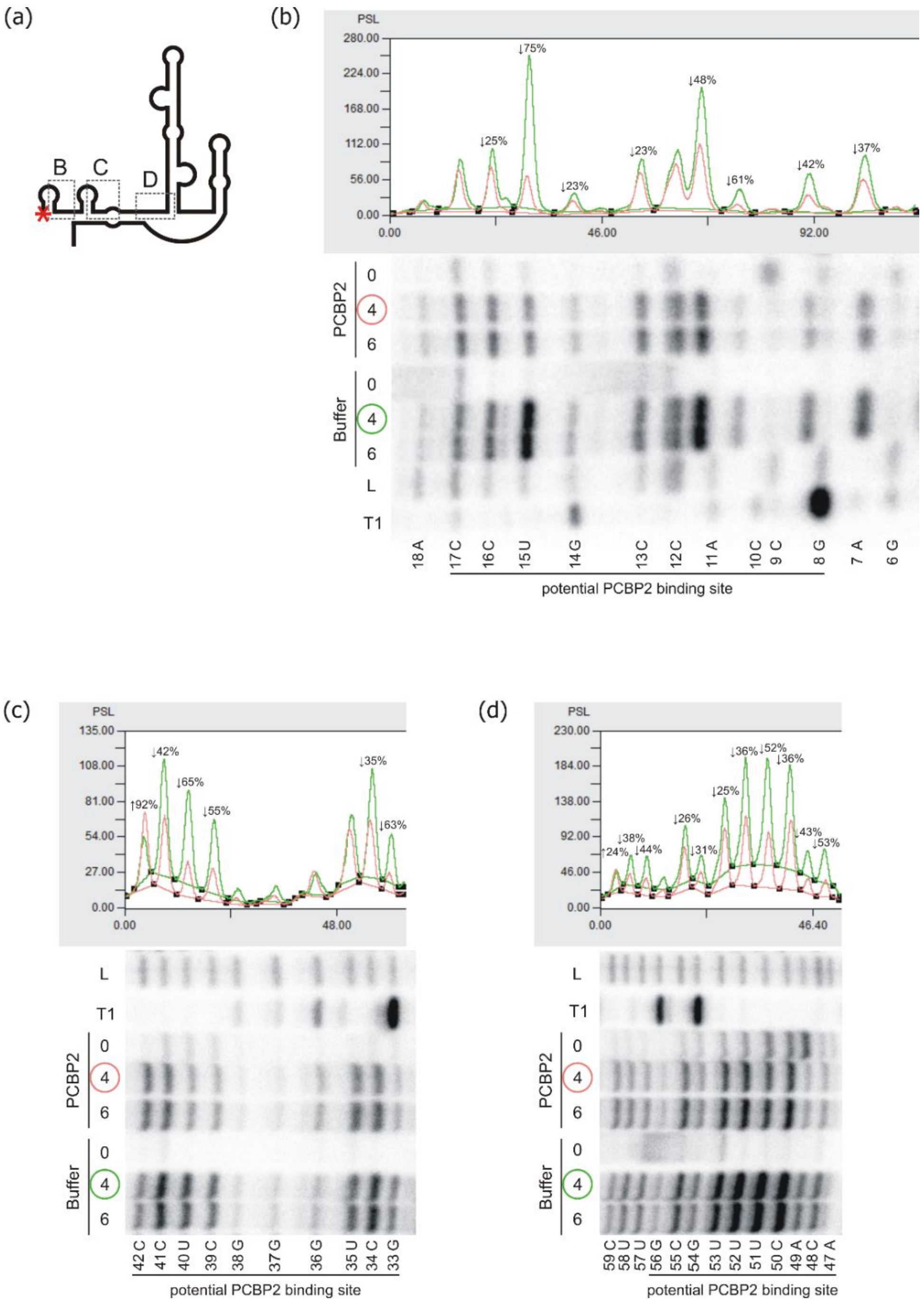

(a)
B
C
D
(b)
PSL
280.00
224.00
168.00
112.00
56.00
0.00
↓75%
↓25%
↓23%
↓23%
↓48%
↓61%
↓42%
↓37%
0.00
46.00
92.00
PCBP2
Buffer
0
4
6
L
T1
18A
17C
16C
15U
14G
13C
12C
11 A
10C
9 C
8 G
7 A
6 G
potential PCBP2 binding site
(c)
PSL
135.00
108.00
81.00
54.00
27.00
0.00
↑92%
↓42%
↓65%
↓55%
↓35%
↓63%
0.00
48.00
L
T1
PCBP2
Buffer
0
4
6
42 C
41 C
40 U
39 C
38 G
37 G
36 G
35 U
34 C
33 G
potential PCBP2 binding site
(d)
PSL
230.00
184.00
138.00
92.00
46.00
0.00
↑24%
↓38%
↓44%
↓26%
↓31%
↓25%
↓36%
↓52%
↓36%
↓43%
↓53%
0.00
46.40
L
T1
PCBP2
Buffer
0
4
6
59 C
58 U
57 U
56 G
55 C
54 G
53 U
52 U
51 U
50 C
49 A
48 C
47 A
potential PCBP2 binding site

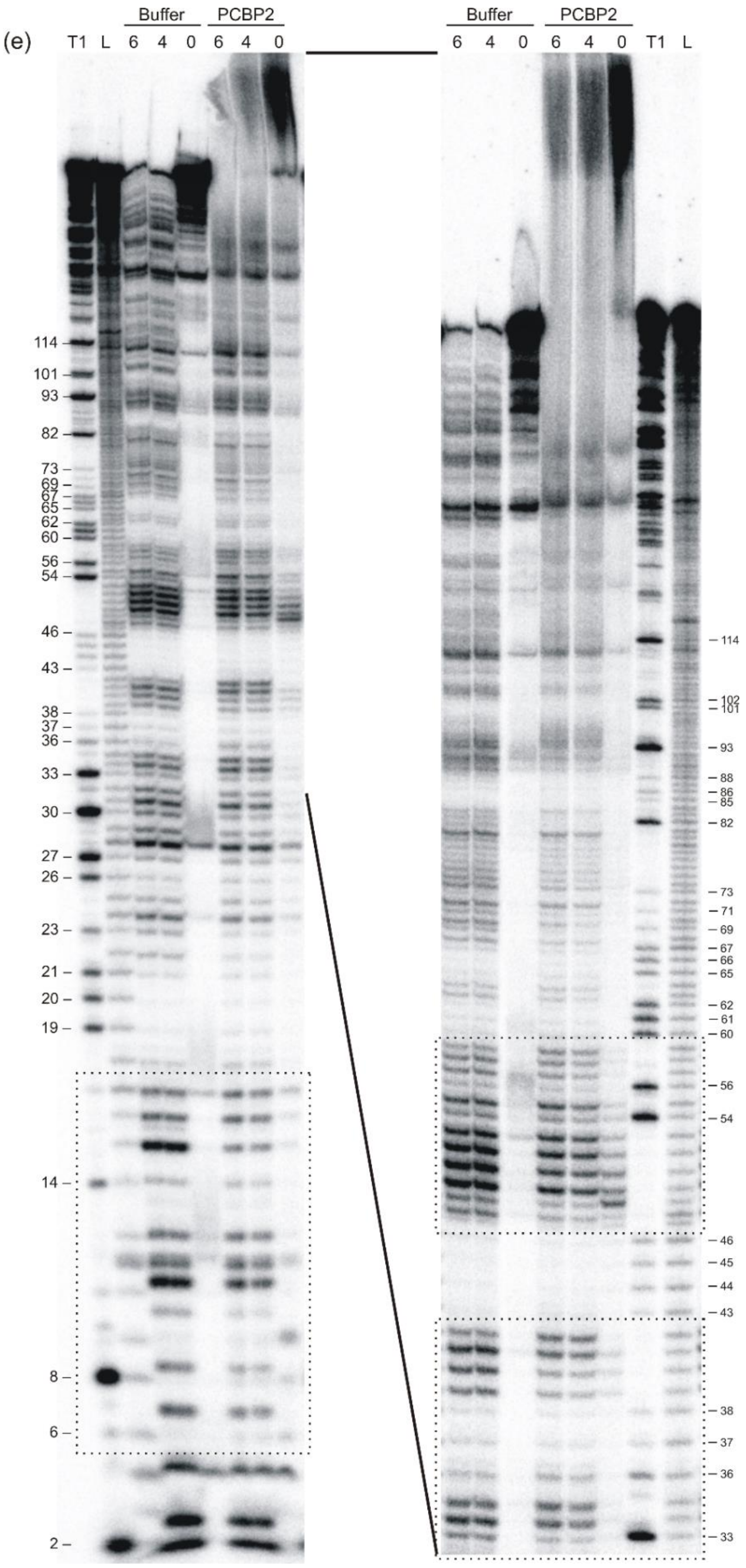
(e)
Buffer
PCBP2
T1 L 6 4 0 6 4 0
6 4 0 6 4 0 T1 L
114
101
93
82
73
69
67
65
62
60
56
54
46
43
38
37
36
33
30
27
26
23
21
20
19
14
8
6
2
114
102
101
93
88
86
85
82
73
71
69
67
66
65
62
61
60
56
54
46
45
44
43
38
37
36
33

Figure S4

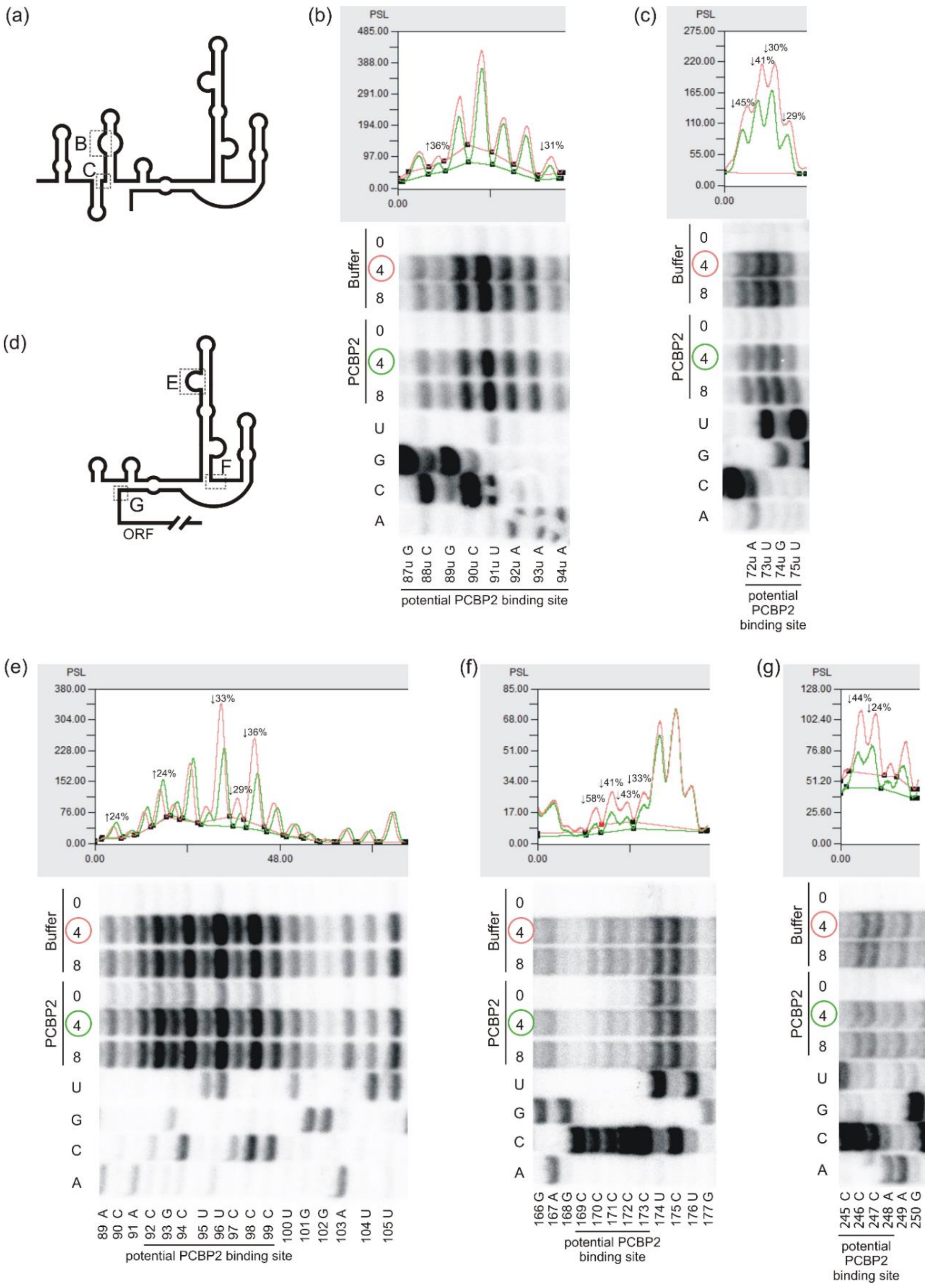

(a)
B
C
(b)
PSL
↑36%
↓31%
Buffer
PCBP2
U
G
C
A
87u G 88u C 89u G 90u C 91u U 92u A 93u A 94u A
potential PCBP2 binding site
(c)
↓45% ↓41% ↓30% ↓29%
72u A 73u U 74u G 75u U
potential PCBP2 binding site
(d)
E
F
G
ORF
(e)
↑24% ↑24% ↓33% ↓29% ↓36%
89 A 90 C 91 A 92 C 93 G 94 C 95 U 96 U 97 C 98 C 99 C 100 U 101 G 102 G 103 A 104 U 105 U
potential PCBP2 binding site
(f)
↓58% ↓41% ↓43% ↓33%
166 G 167 A 168 G 169 C 170 C 171 C 172 C 173 C 174 U 175 C 176 U 177 G
potential PCBP2 binding site
(g)
↓44% ↓24%
245 C 246 C 247 C 248 A 249 A 250 G
potential PCBP2 binding site

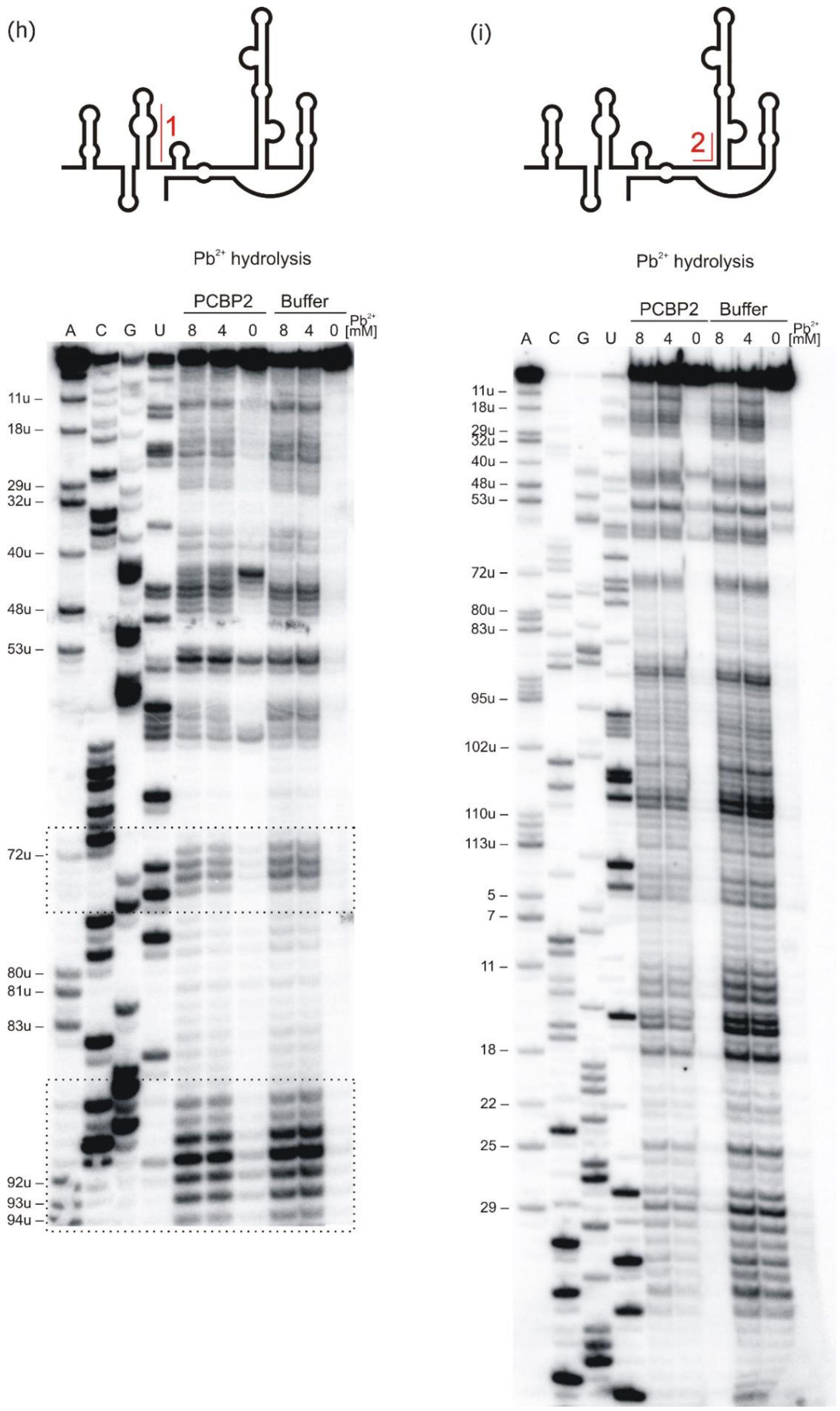

(h)
1
Pb2+ hydrolysis
PCBP2
Buffer
A C G U 8 4 0 8 4 0
Pb2+ [mM]
11u
18u
29u
32u
40u
48u
53u
72u
80u
81u
83u
92u
93u
94u
(i)
2
Pb2+ hydrolysis
PCBP2
Buffer
A C G U 8 4 0 8 4 0
Pb2+ [mM]
11u
18u
29u
32u
40u
48u
53u
72u
80u
83u
95u
102u
110u
113u
5
7
11
18
22
25
29

(j)

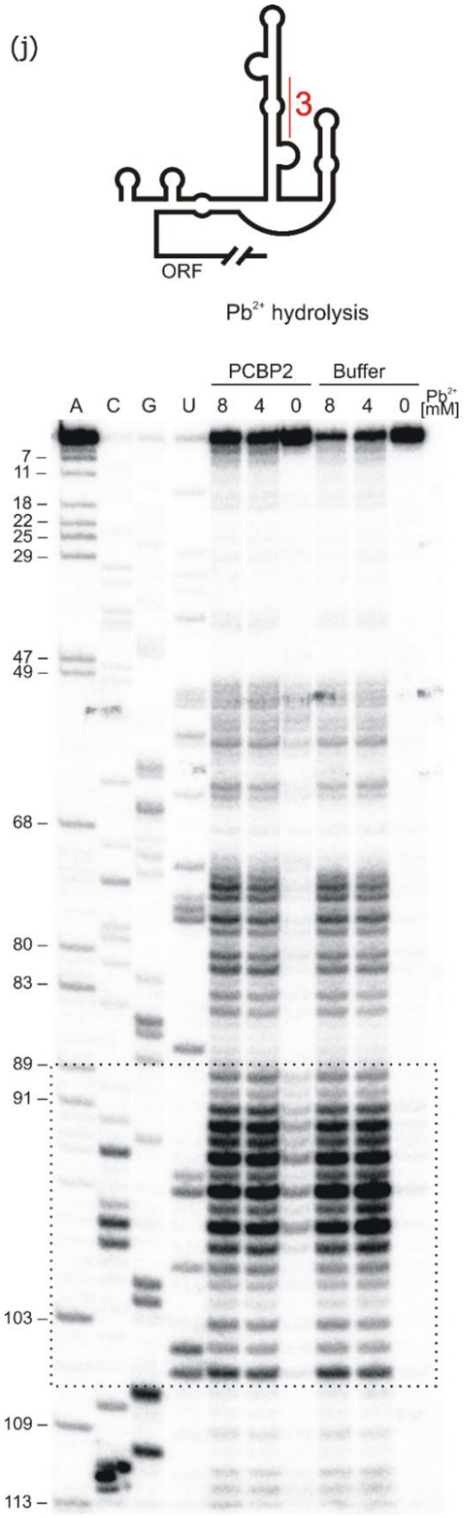
3
ORF
Pb2+ hydrolysis
PCBP2
Buffer
A C G U 8 4 0 8 4 0 Pb2+ [mM]
7
11
18
22
25
29
47
49
68
80
83
89
91
103
109
113

(k)

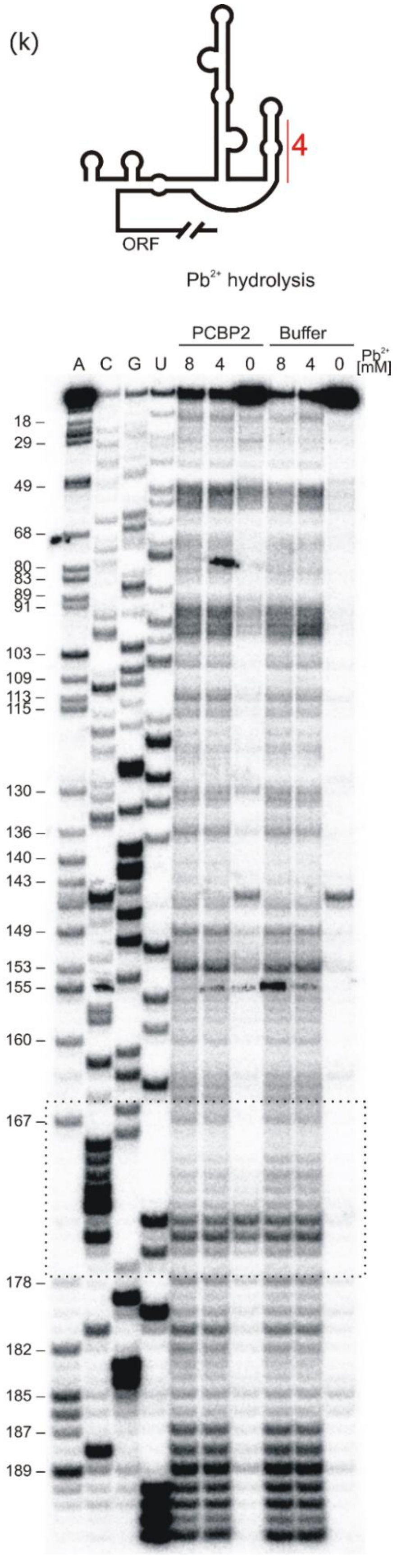
4
ORF
Pb2+ hydrolysis
PCBP2
Buffer
A C G U 8 4 0 8 4 0 Pb2+ [mM]
18
29
49
68
80
83
89
91
103
109
113
115
130
136
140
143
149
153
155
160
167
178
182
185
187
189

(I)

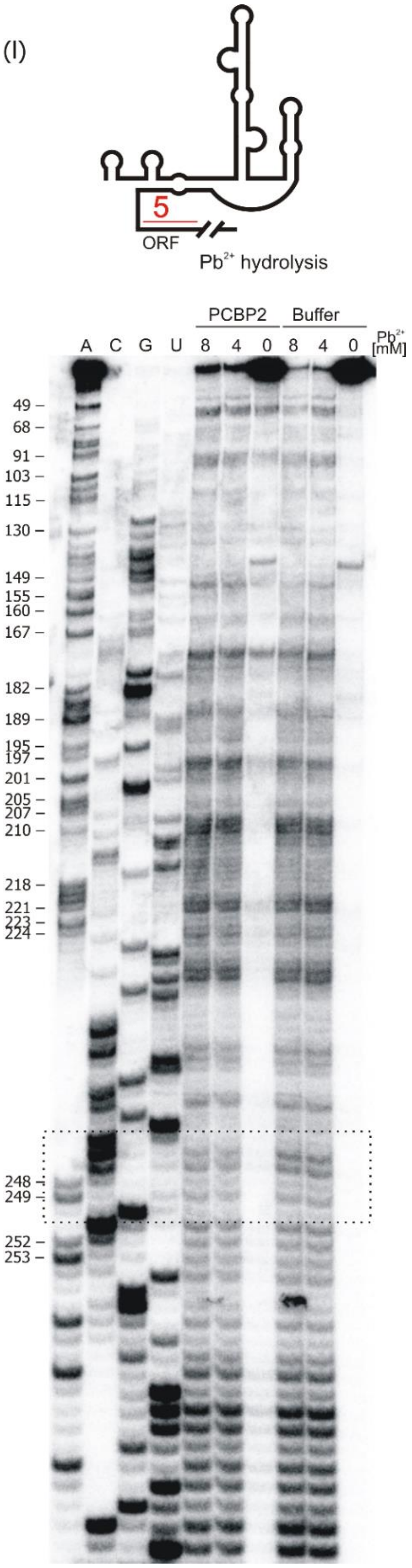

5
ORF
Pb²⁺ hydrolysis
PCBP2
Buffer
A C G U 8 4 0 8 4 0
Pb²⁺ [mM]
49
68
91
103
115
130
149
155
160
167
182
189
195
197
201
205
207
210
218
221
223
224
248
249
252
253

Figure S5

(a)

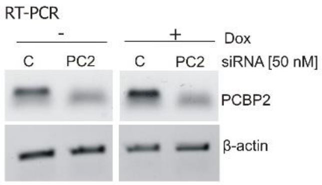
RT-PCR
-
+
Dox
C
PC2
C
PC2
siRNA [50 nM]
PCBP2
β-actin

(b)

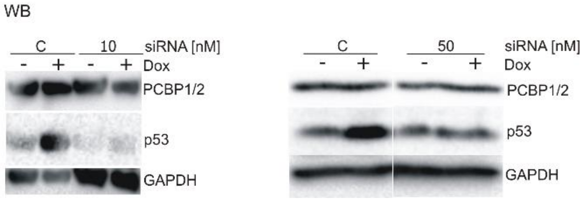
WB
C
10
siRNA [nM]
- + - +
Dox
PCBP1/2
p53
GAPDH
C
50
siRNA [nM]
- + - +
Dox
PCBP1/2
p53
GAPDH
